# An Optogenetic Method to Modulate Cell Contractility during Tissue Morphogenesis

**DOI:** 10.1016/j.devcel.2015.10.020

**Published:** 2015-12-07

**Authors:** Giorgia Guglielmi, Joseph D. Barry, Wolfgang Huber, Stefano De Renzis

**Affiliations:** 1 EMBL Heidelberg, Meyerhofstrasse 1, 69117 Heidelberg, Germany

## Abstract

Morphogenesis of multicellular organisms is driven by localized cell shape changes. How, and to what extent, changes in behavior in single cells or groups of cells influence neighboring cells and large-scale tissue remodeling remains an open question. Indeed, our understanding of multicellular dynamics is limited by the lack of methods allowing the modulation of cell behavior with high spatiotemporal precision. Here, we developed an optogenetic approach to achieve local modulation of cell contractility and used it to control morphogenetic movements during *Drosophila* embryogenesis. We show that local inhibition of apical constriction is sufficient to cause a global arrest of mesoderm invagination. By varying the spatial pattern of inhibition during invagination, we further demonstrate that coordinated contractile behavior responds to local tissue geometrical constraints. Together, these results show the efficacy of this optogenetic approach to dissect the interplay between cell-cell interaction, force transmission, and tissue geometry during complex morphogenetic processes.

## Highlights

• Optogenetics provides a powerful approach to control tissue morphogenesis
• Two-photon illumination allows precise patterns of optogenetic activation
• Local modulation of cell contractility reveals mechanisms of tissue invagination
• Tissue geometry constrains the cell contractile behavior driving invagination

## Graphical Abstract

**Figure:**
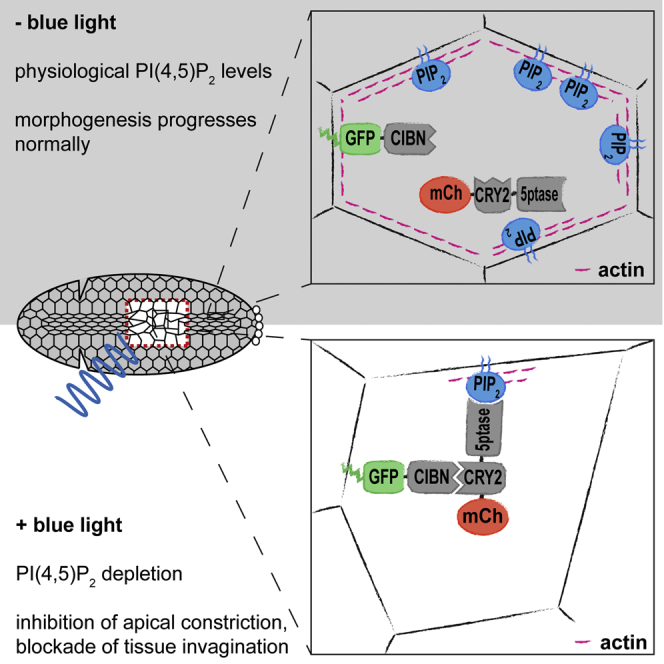

## Teaser

How localized changes in cell behavior influence global tissue remodeling remains an open question. In this Resource article, Guglielmi et al. present an optogenetic approach to locally modulate cell contractility during *Drosophila* embryogenesis and use it to probe cell-cell interaction, force transmission, and tissue geometry during mesoderm invagination.

## Introduction

During embryonic development, tissue remodeling results from local cell shape changes, often initiated by the activation and association of non-muscle myosin II with actin filaments (Behrndt et al., 2012, Bertet et al., 2004, Mayer et al., 2010, Zallen and Wieschaus, 2004). Tension generated by actomyosin contractility can induce changes in cell morphology, such as apical constriction (Sherrard et al., 2010), and can be transmitted to other cells via adherens junctions, generating more complex responses at the tissue level (Kölsch et al., 2007, Lecuit et al., 2011, Martin and Goldstein, 2014, Sadler et al., 1982). How cell-cell interaction can give rise to a wide range of different morphogenetic movements is an area of active investigation. Open questions relate to the spatial extent of force transmission necessary to drive collective behaviors and the degree to which neighboring tissues influence each other’s dynamics (Heisenberg and Bellaïche, 2013). Furthermore, given that multiple morphogenetic processes occur simultaneously in a closed environment, predefined spatial constraints are also likely to impact on tissue dynamics.

To address these questions, it would be advantageous to interfere with specific cell behaviors in single cells or multiple cells simultaneously. Although traditional genetic approaches such as knockouts, knockdowns, mutations, and overexpression of specific proteins have led to the discovery of key molecular mechanisms regulating cell and tissue shape (Irvine and Wieschaus, 1994, Leptin and Roth, 1994, Pelissier et al., 2003), they do not provide efficient means to manipulate cell activity with high spatiotemporal precision. To overcome this limitation, new chemical tools have been developed (Putyrski and Schultz, 2012). However, these approaches still lack spatial specificity and, when used in living organisms, often require microinjection, which can potentially result in tissue damage. More recently, laser ablation has emerged as a useful technique to perturb single cells in the context of developing organisms (Martin et al., 2010, Rauzi et al., 2008). Ablation of biological structures through high laser intensity elicits fast responses with high spatial precision but can lead to unwanted cytotoxic effects.

Optogenetics is an emerging technique that allows the control of cell activity by exploiting genetically encoded photo-activatable proteins or protein modules. It combines the advantages of chemical tools in terms of flexibility in the responses that can be elicited (i.e., both activation and inhibition of protein function) with the pros of laser ablation with regard to spatial and temporal specificity. Also, since the laser energies and wavelengths used to stimulate photo-activatable modules are in the same range as those used for optical imaging, cytotoxicity is reduced to minimal, if not negligible, levels (Toettcher et al., 2011). Optogenetics might be, therefore, an excellent technique to modulate cell activity during organismal development.

In this report, we describe an optogenetic method that allows precise spatiotemporal modulation of cell contractility during tissue morphogenesis. Given the essential role played by plasma membrane phosphoinositides in regulating actin polymerization at specific plasma membrane domains (Bardet et al., 2013, Comer and Parent, 2007, Janetopoulos and Devreotes, 2006, Reversi et al., 2014, Rozelle et al., 2000), we sought to modulate the levels of these lipid species—in particular, of phosphatidylinositol-4,5 bisphosphate (PI(4,5)P_2_)—to achieve control over cortical actin polymerization and cell contractility. We illustrate the validity of this optogenetic approach by focusing on ventral furrow formation, the morphogenetic process that leads to the internalization of the presumptive mesoderm during *Drosophila* embryogenesis (Kölsch et al., 2007, Martin et al., 2009, McMahon et al., 2008, Sweeton et al., 1991). During ventral furrow formation, a group of approximately 1,000 cells organized in a rectangular conformation along the embryonic anterior-posterior (a-p) axis starts constricting apically and then invaginates (Sweeton et al., 1991). Coupling of cell contractility among neighboring ventral cells is thought to generate a tissue-level force, which, in turn, facilitates invagination (Martin et al., 2010). However, the spatial range of force integration necessary to drive collective contractions and tissue invagination remains difficult to address with current tools. Furthermore, the extent to which apical constriction is required for invagination is controversial, as computational models suggest a requirement for additional pushing forces by lateral ectodermal cells (Conte et al., 2009, Conte et al., 2012). By combining single- and two-photon laser illumination with quantitative imaging, our results demonstrate that optogenetic regulation of PI(4,5)P_2_ plasma membrane levels allows the modulation of apical constriction with spatial (cellular) and temporal (seconds) precision, thus providing a powerful method to dissect the interplay between cell-cell interaction, force transmission, and tissue geometry during tissue morphogenesis.

## Results

### Light-Mediated CRY2-OCRL Plasma Membrane Recruitment Results in PI(4,5)P_2_ and Actin Depletion from the Embryo Cortex

We used the Cryptochrome2 (CRY2)-CIB1 protein dimerization system to control the localization of the catalytic domain of the inositol polyphosphate 5-phosphatase OCRL, which converts PI(4,5)P_2_ into phosphatidylinositol-4-phosphate (PI(4)P) (Zhang et al., 1995). This approach has been shown to allow quick depletion of PI(4,5)P_2_ in cell culture (Giordano et al., 2013, Idevall-Hagren et al., 2012); therefore, given the essential role of PI(4,5)P_2_ plasma membrane levels in controlling cortical actin dynamics (Bardet et al., 2013, Comer and Parent, 2007, Janetopoulos and Devreotes, 2006, Reversi et al., 2014, Rozelle et al., 2000), it might provide an efficient means to modulate cell contractility during tissue morphogenesis.

**Figure 1:**
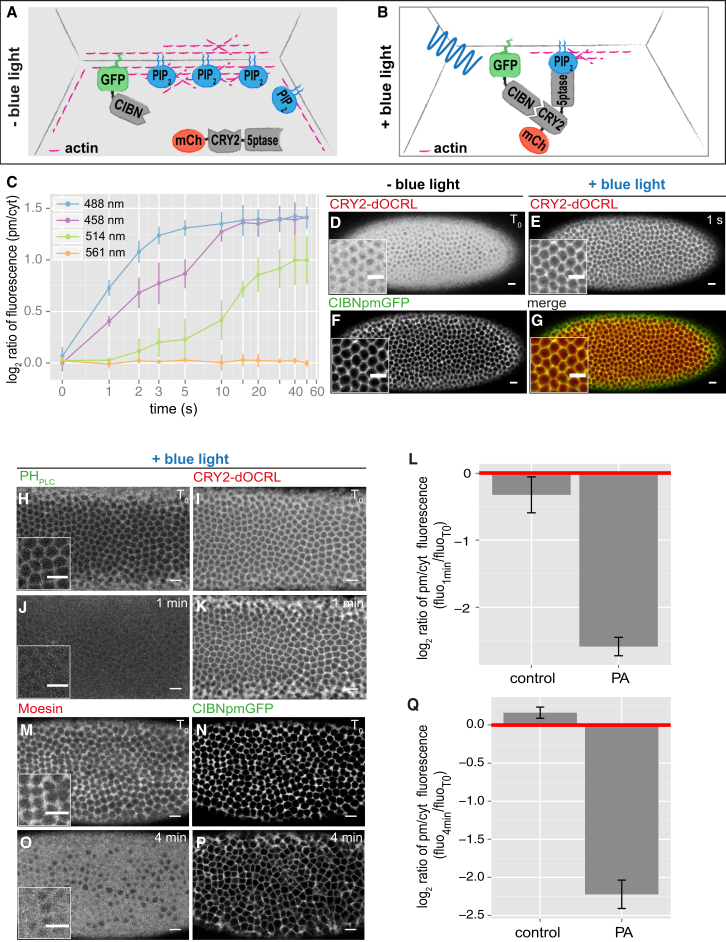
Light-Mediated CRY2-OCRL Plasma Membrane Recruitment Results in PI(4,5)P_2_ and Actin Depletion from the Embryo Cortex (A and B) Cartoon depicting a surface view of the *Drosophila* blastoderm epithelium and schematic of the optogenetic module that we used. This is based on the interaction between CIBN and the PHR domain of CRY2 upon blue-light (488-nm) illumination. CIBN was tagged with enhanced GFP and with a CaaX anchor, which localizes it to the plasma membrane. CRY2 was fused to the catalytic domain of the *Drosophila* inositol polyphosphate 5-phosphatase OCRL and tagged with mCherry. In the absence of blue light, the 5-ptase is cytosolic (A). Upon blue-light illumination, the 5-ptase is recruited to the plasma membrane, where it dephosphorylates PI(4,5)P_2_ to PI(4)P. This results in PI(4,5)P_2_ depletion from the plasma membrane and cortical actin depolymerization (B). (C) Mean levels of mCherry::CRY2-OCRL recruitment to the plasma-membrane-anchored CIBN::pmGFP in response to 488-, 458-, 514-, and 561-nm light. The x coordinates represent time of exposure to light. Photo-activation was achieved using a continuous-wave laser of the indicated wavelength at a scanning speed of 1.27 × 10^−6^ s/pixel, for a total time of 1 s for the entire embryo at 30-s intervals. The y coordinates represent the log_2_ ratio of CRY2-OCRL plasma membrane (pm) to cytosol (cyt) fluorescence intensities. Pooled data are represented as mean ± SD (n = 3 embryos for each condition). (D–G) Confocal images of a representative embryo co-expressing CIBN::pmGFP and mCherry::CRY2-OCRL. Before blue-light illumination, mCherry::CRY2-OCRL is cytosolic (D). After 1-s exposure to 488-nm light, mCherry::CRY2-OCRL is recruited to the plasma membrane (E), where CIBN::pmGFP localizes (F). (G) shows a merge of (E and F). Scale bars, 10 μm. (H–K) Confocal images of a representative embryo co-expressing a non-tagged version of CIBN, the PI(4,5)P_2_ biosensor PH_PLCδ_::GFP, and mCherry::CRY2-OCRL. PH_PLCδ_::GFP is depleted from the plasma membrane upon illumination at 488 nm: compare signal (H) at the onset of photo-activation (T_0_) and (J) 1 min after the beginning of photo-activation. Of note, the total signal intensities shown in (H) and (J) are approximately the same (3.663 versus 3.531, a.u.). (I and K) show the localization of mCherry::CRY2-OCRL at the onset (T_0_) and at 1 min after the beginning of photo-activation, respectively. Scale bars, 10 μm. (L) Barplot showing mean PH_PLCδ_::GFP levels in control and photo-activated (PA) embryos. Control embryos express mCherry::CRY2-OCRL and PH_PLCδ_::GFP. PA embryos co-express a non-tagged version of CIBN (to avoid overlap with PH_PLCδ_::GFP signal), PH_PLCδ_::GFP, and mCherry::CRY2-OCRL. Both control and PA embryos were exposed to 488-nm illumination. Values along the y axis represent the log_2_ ratio of the average PH_PLCδ_::GFP plasma membrane fluorescence intensity at 1 min after the beginning of photo-activation (fluo_1min_) to the average PH_PLCδ_::GFP plasma membrane fluorescence intensity at the beginning of photo-activation (fluo_T0_). Pooled data are represented as mean ± SD (n = 3 embryos for each condition; p = 1.0 × 10^−3^; two-sample Student’s t test). (M–P) Confocal images of a representative embryo co-expressing a non-tagged version of CRY2-OCRL, the actin-binding protein Moesin::mCherry, and CIBN::pmGFP. Moesin::mCherry localizes to the cell cortex at the beginning (T_0_) of photo-activation (M) and is depleted 4 min after the beginning of photo-activation (O). (N and P) show the localization of CIBN::pmGFP at the beginning (T_0_) and 4 min after the beginning of photo-activation, respectively. Scale bars, 10 μm. (Q) Barplot showing mean Moesin::mCherry levels in control and photo-activated (PA) embryos. Control embryos express CIBN::pmGFP and Moesin::mCherry. PA embryos co-express a non-tagged version of CRY2-OCRL (to avoid overlap with Moesin::mCherry signal), Moesin::mCherry, and CIBN::pmGFP. Both control and PA embryos were exposed to 488-nm light. The y axis displays the log_2_ ratio of the average Moesin::mCherry plasma membrane fluorescence intensity at 4 min after the beginning of photo-activation (fluo_4min_) to the average Moesin::mCherry plasma membrane fluorescence intensity at the beginning of photo-activation (fluo_T0_). Pooled data are represented as mean ± SD (n = 3 embryos for each condition; p = 5.7 × 10^−4^; two-sample Student’s t test). See also Figure S1.

The CRY2-CIB1 protein dimerization system is based on the interaction between the N-terminal end of CIB1 (CIBN) and the PHR domain of CRY2 upon blue-light illumination (Liu et al., 2008). It allows rapid (subsecond-scale) activation, and it does not require the addition of an exogenous chromophore (Kennedy et al., 2010). We generated *Drosophila* embryos expressing both the plasma membrane anchor CIBN::pmEGFP (CIBN fused to a version of enhanced GFP that localizes to the plasma membrane) and the photoreactive domain of CRY2 fused to the catalytic domain of the *Drosophila* inositol polyphosphate 5-phosphatase OCRL tagged with mCherry, hereinafter referred to as CRY2-OCRL (Figures 1A and 1B).

To test whether the CRY2-CIB1 system could be used for in vivo application, we measured the level of CRY2-OCRL plasma membrane recruitment at different wavelengths (Figure 1C). Consistent with the activation peak of the CRY2-CIB1 system measured in cell culture (Kennedy et al., 2010), illumination at 488 nm using a continuous-wave argon laser was the most effective way to translocate CRY2-OCRL from the cytosol, where it localizes in the dark, to the plasma membrane, where the CIBN anchor is located (Figures 1D–1G). The illumination time (t_1/2_) necessary for the translocation to occur was 1.27 × 10^−6^ s/pixel, corresponding to around 2.1 ms per cell and 1 s for the entire embryo. Illumination at 458-nm and 514-nm light resulted in lower efficiencies and slower kinetics of activation (t_1/2_ = 3 s and 12.5 s, respectively; see purple and green lines in Figure 1C), while illumination at 561 nm did not result in CRY2-OCRL plasma membrane recruitment, arguing that, at this wavelength, CRY2 is not activated. Importantly, CRY2-OCRL plasma membrane recruitment depended on the co-expression of the CIBN anchor and resulted in the depletion of PI(4,5)P_2_ and actin within 1 min and 4 min of 488-nm light exposure, respectively, as demonstrated by the plasma membrane dissociation of the PI(4,5)P_2_ biosensor PH_PLCδ_-GFP (Figures 1H–1L) and of the actin-binding protein Moesin (Figures 1M–1Q). The association of CRY2-OCRL with the plasma-membrane-anchored CIBN was reversible, with a t_1/2_ of ∼8.9 min at room temperature (Figure S1A). Similarly, the dissociation of Moesin from the plasma membrane reverted with a t_1/2_ of ∼7.5 min (Figures S1B–S1E). Taken together, these results show that CRY2-OCRL plasma membrane recruitment upon a short exposure (second scale) to 488-nm light provides a rapid way to deplete PI(4,5)P_2_ and, in turn, actin from the cell cortex of the embryo. Consistently, light-mediated translocation of CRY2-OCRL to the plasma membrane resulted in the arrest of cellularization (Figures S1F–S1I), an actin-dependent morphogenetic process that transforms the syncytial embryo into 6,000 mononucleated epithelial cells (Harris et al., 2009). This phenotype precisely recapitulates the morphological effects induced upon chemical depletion of PI(4,5)P_2_ or inhibition of actin polymerization using cytochalasin D (Reversi et al., 2014). Importantly, embryos expressing only the plasma membrane anchor CIBN::pmGFP showed normal cellularization dynamics (Figures S1J–S1L).

### Activation of CRY2-OCRL Plasma Membrane Recruitment Causes Inhibition of Apical Constrictions and Arrest of Ventral Furrow Formation

Having established a method to disrupt an actin-mediated morphogenetic event, we wondered whether it could modulate cell constriction to the point at which we could disrupt ventral furrow formation. During this process, a group of epithelial cells along the ventral midline constricts apically and eventually invaginates, forming a tube inside the embryo. The apical recruitment of non-muscle myosin II (myosin) on actin filaments initiates contractions in ventral cells. The force generated by myosin motor is propagated to neighboring cells through apically anchored adherens junctions, whose cytosolic side is linked to the actin cytoskeleton. During ventral furrow invagination, cell constriction is orthogonal to the embryonic a-p axis. This means that cells reduce their size preferentially in a direction that is perpendicular to the a-p axis, acquiring an eccentric morphology. This type of behavior is known as a-p anisotropic constriction (Martin et al., 2009, Martin et al., 2010).

**Figure 2:**
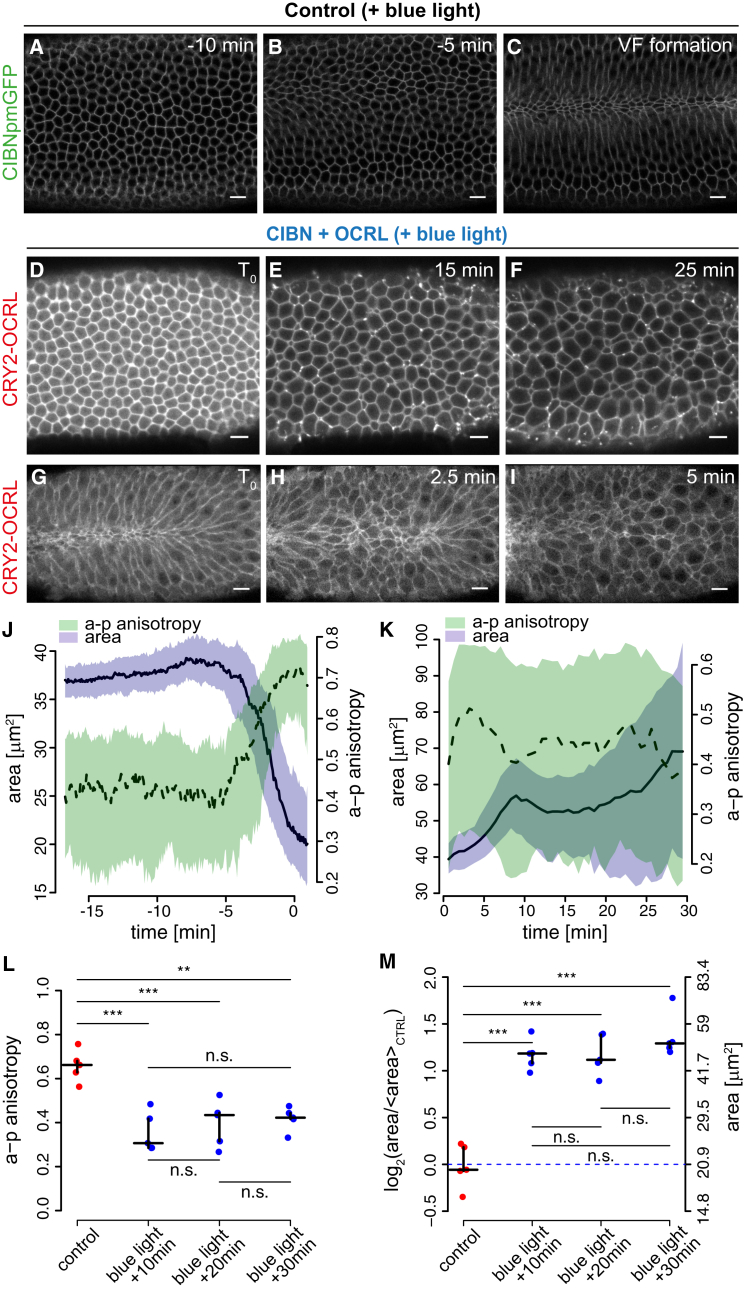
Activation of CRY2-OCRL Plasma Membrane Recruitment Causes Inhibition of Apical Constrictions and Arrest of Ventral Furrow Formation (A–C) Still frames from a confocal movie of the ventral mesoderm of a representative control embryo expressing only CIBN::pmGFP 10 min before ventral furrow formation (A), 5 min before ventral furrow (VF) formation (B), and at the onset of ventral furrow formation (C). Scale bars, 10 μm. (D–F) Still frames from a confocal movie of the ventral mesoderm of a representative embryo co-expressing CIBN::pmGFP and mCherry::CRY2-OCRL at the onset of photo-activation (T_0_) (D) and at 15 min (E) and 25 min (F) after the beginning of photo-activation. Photo-activation was started before the beginning of ventral furrow formation using a continuous 488-nm wave laser at a scanning speed of 1.27 × 10^−6^ s/pixel, for a total time of 1 s for the entire embryo at 30-s intervals. Scale bars, 10 μm. (G–I) Still frames from a confocal movie of the ventral mesoderm of a representative embryo co-expressing CIBN::pmGFP and mCherry::CRY2-OCRL at the beginning of photo-activation (T_0_) (G) and at 2.5 min (H) and 5 min (I) after the beginning of photo-activation. Photo-activation was started after the beginning of ventral furrow formation and resulted in the reversion of the invagination process with alternated patches of constricted and relaxed cells. Scale bars, 10 μm. (J and K) Quantification of cell area (purple) and a-p anisotropy (green) for (J) the control embryo shown in (A–C) and for (K) the photo-activated embryo expressing CRY2-OCRL shown in (D–F). The y coordinates represent cell area expressed in squared microns (left y axis) and a-p anisotropy (right y axis). Values along the x axis represent time in minutes. Solid and dashed lines indicate the median over all cells for cell area (solid) and a-p anisotropy (dashed). Shaded regions show the interquartile range. In (J), time 0 corresponds to the time point of ventral furrow invagination. In (K), time 0 corresponds to the beginning of photo-activation. (L and M) Comparison of a-p anisotropy (L) and cell area (M) between control embryos expressing only CIBN::pmGFP (at the time point of tissue invagination) and photo-activated embryos co-expressing CIBN::pmGFP and mCherry::CRY2-OCRL at three different time points after the beginning of photo-activation (10 min, 20 min, and 30 min). Values along the y axis represent a-p anisotropy in (L) and cell area in (M). Statistical testing of differences in cell area was performed on the log-transformed values (left y axis). Absolute values for cell areas (in squared microns) are represented on the right y axis as a reference (M). For both cell area and a-p anisotropy, the control samples are significantly different from the photo-activated samples. Each dot represents a single embryo. The crosses show group median (horizontal line) and interquartile range (vertical line). (n = 5 embryos for each condition). ^∗^p ≤ 0.05; ^∗∗^p ≤ 0.01; ^∗∗∗^p ≤ 0.001; n.s., not significant; pairwise two-sample Student’s t tests with pooled variance and multiple testing correction with Bonferroni’s method. See also Movies S1 and S2A and S2B.

**Figure 3:**
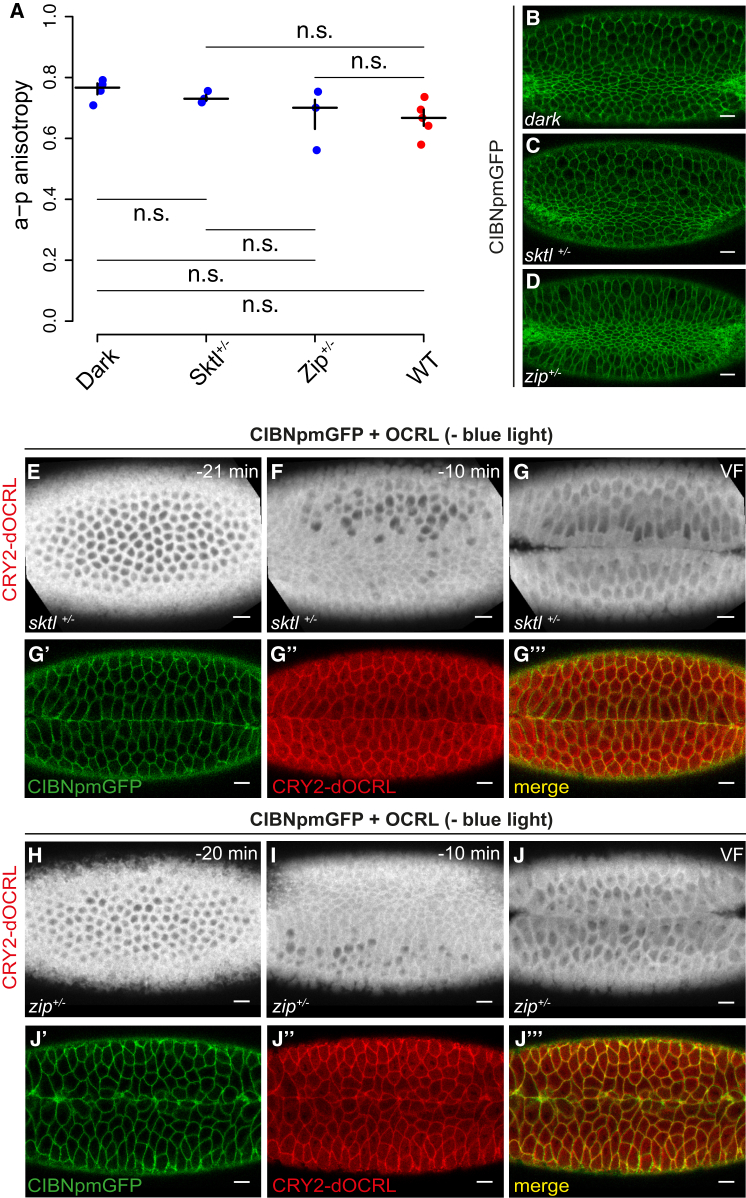
Ventral Furrow Formation Is Not Inhibited in Embryos Co-expressing CRY2-OCRL and CIBN::pmGFP in the Absence of 488-nm Illumination (A) Comparison of median a-p anisotropy of constricting cells in the absence of blue-light illumination between embryos co-expressing CIBN::pmGFP and mCherry::CRY2-OCRL in a WT background (Dark); embryos co-expressing CIBN::pmGFP and mCherry::CRY2-OCRL in a *sktl* (Sktl^+/−^) and in a *zip* (Zip^+/−^) heterozygous mutant background; and embryos expressing CIBN::pmGFP only in a WT background (WT). Values along the y axis represent a-p anisotropy. Each dot represents a single embryo. The crosses show group median and interquartile ranges (n ≥ 3 embryos for each condition). n.s., not significant; pairwise two-sample Student’s t tests with pooled variance and multiple testing correction with Bonferroni’s method. (B–D) Confocal images of the ventral mesoderm of representative embryos co-expressing CIBN::pmGFP and mCherry::CRY2-OCRL in a WT background (B) and in a *sktl* (C) and a *zip* (D) heterozygous mutant background. The images were taken with 488-nm light after ventral cells started to constrict. CIBN::pmGFP is shown. Scale bars, 10 μm. (E–G’’’) Still frames from a time-lapse confocal movie of the ventral mesoderm of a representative embryo heterozygous for a loss-of-function *sktl* allele (*sktl*^*+/−*^) and co-expressing CIBN::pmGFP and mCherry::CRY2-OCRL 21 min before (E), 10 min before (F), and at completion of (G) ventral furrow (VF) invagination. The embryo was imaged with 561-nm light only. (G’) shows CIBN::pmGFP, (G’’) shows mCherry::CRY2-OCRL, and (G’’’) shows a merge of the two channels at completion of ventral furrow invagination. Scale bars, 10 μm. (H–J’’’) Still frames from a time-lapse confocal movie of the ventral mesoderm of a representative embryo heterozygous for a loss-of-function *zip* allele (*zip*^*+/−*^) and co-expressing CIBN::pmGFP and mCherry::CRY2-OCRL 20 min before (H), 10 min before (I), and at completion of (J) ventral furrow invagination. The embryo was imaged with 561-nm light only. (J’) shows CIBN::pmGFP, (J’’) shows mCherry::CRY2-OCRL, and (J’’’) shows a merge of the two channels at completion of ventral furrow invagination. Scale bars, 10 μm. See also Figure S2 and Movies S3A and S3B.

First, we tested whether modulation of PI(4,5)P_2_ levels would provide an efficient means to disrupt cell contractility by triggering CRY2-OCRL plasma membrane recruitment in the whole embryo. Global activation of CRY2-OCRL plasma membrane recruitment resulted in the arrest of ventral furrow formation if the pulse of light was given before cells started to constrict and in the reversion of invagination if the pulse of light was given when cells were already constricted (Figures 2A–2I; Movie S1; Movies S2A and S2B). In agreement with previous reports (Martin et al., 2009, Martin et al., 2010), quantification of surface area in control embryos showed that ventral cells constrict in an a-p anisotropic fashion (Figure 2J). CRY2-OCRL plasma membrane recruitment caused both inhibition of apical constriction and loss of a-p anisotropy (Figures 2K–2M). This demonstrates that modulation of PI(4,5)P_2_ levels provides a powerful tool to control apical constriction during invagination. Importantly, overexpression of CRY2-OCRL alone did not have any effect on the invagination process (Figures S2A–S2C), and embryos co-expressing CRY2-OCRL and CIBN::pmGFP did not show any abnormalities when imaged at 561 nm, a wavelength that does not trigger CRY2-OCRL plasma membrane recruitment and that we, therefore, define as “dark.” Indeed, these embryos showed normal a-p anisotropic constrictions and ventral furrow kinetics comparable to those of wild-type (WT) embryos (Figures 3A and 3B; Figures S2D–S2F’’’). Lastly, we followed ventral furrow formation in embryos heterozygous for either *zip* or *sktl* loss-of-function alleles, which co-expressed CRY2-OCRL and CIBN::pmGFP. *zip* (*zipper*) and *sktl* (*skittles*) encode for myosin heavy chain and for phosphatidylinositol 4-phosphate 5 kinase (PIP5K), respectively. Because myosin and PI(4,5)P_2_ are required for apical constriction, we reasoned that a reduction in the activity of either myosin or PIP5K (which converts PI(4)P into PI(4,5)P_2_) should uncover any potential dark-state interaction between CRY2-OCRL and CIBN::pmGFP. As shown in Figure 3, both a-p anisotropic constrictions and ventral furrow kinetics were preserved when *zip* or *sktl* heterozygous embryos co-expressing CRY2-OCRL and CIBN::pmGFP were imaged at 561 nm (Figures 3A and 3C–3J’’’; Movies S3A and S3B). These experiments show that this optogenetic approach is silent in the dark (i.e., CRY2-OCRL and CIBN::pmGFP only interact in the presence of blue light) and, therefore, that the effects on apical constriction are due exclusively to light-mediated activation of CRY2-OCRL plasma membrane recruitment.

### Two-Photon Laser Activation Allows Modulation of Apical Constriction with Spatial Precision

**Figure 4:**
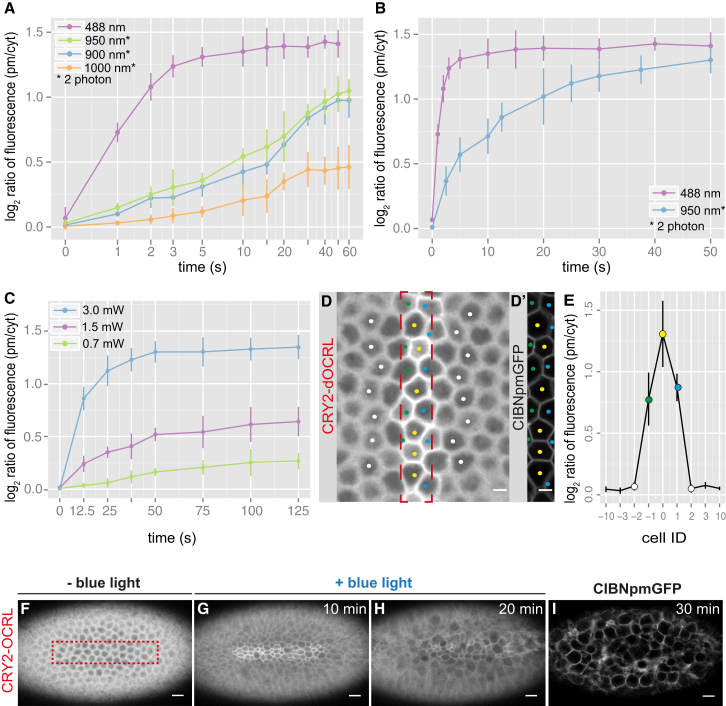
Two-Photon Activation Allows Local Modulation of Cell Contractility (A) Mean levels of mCherry::CRY2-OCRL plasma membrane recruitment in response to 488-nm light (one-photon illumination) and to 900-, 950-, and 1,000-nm light; two-photon illumination, achieved using a femtosecond (140-fs) pulsed laser at a repetition rate of 80 MHz. A single z stack was illuminated 5 μm from the apical surface, for a total scanning time of 1 s. In between two consecutive scans, there was an interval of 30 s and the imaging of mCherry::CRY2-OCRL at 561 nm. The protocol for 488-nm illumination is the same as described in Figure 1C. The values on the x axis correspond to the time of two-photon or 488-nm illumination. The y coordinates represent the log_2_ ratio of mCherry::CRY2-OCRL plasma membrane (pm) to cytosol (cyt) fluorescence intensities. Pooled data are represented as mean ± SD (n = 3 embryos for each condition). (B) Mean levels of mCherry::CRY2-OCRL plasma membrane recruitment in response to 488- and 950-nm light. For λ = 488 nm, a single z stack was illuminated for 1 s. For λ = 950 nm, five consecutive z stacks separated by 1 μm were illuminated for ∼500 ms, for a total scan time of 2.5 s. In between two consecutive pulses, there was an interval of 30 s and the imaging of mCherry::CRY2-OCRL at 561 nm. The values on the x axis correspond to the time of two-photon or 488-nm illumination. The y axis displays the log_2_ ratio of mCherry::CRY2-OCRL plasma membrane to cytosol fluorescence intensities. Pooled data are represented as mean ± SD (n = 3 embryos for each condition). (C) Mean levels of mCherry::CRY2-OCRL plasma membrane recruitment in response to excitation with a 950-nm laser at three different powers (3.0 mW, 1.5 mW, and 0.7 mW). Photo-activation was achieved by illuminating five consecutive z stacks separated by 1 μm for ∼500 ms each (total scan time = 2.5 s). In between two consecutive scans, there was an interval of 30 s and the imaging of mCherry::CRY2-OCRL at 561 nm. The values on the x axis correspond to the time of two-photon illumination. The y coordinates represent the log_2_ ratio of mCherry::CRY2-OCRL plasma membrane to cytosol fluorescence intensity. Pooled data are represented as mean ± SD (n = 3 embryos for each condition). (D) Confocal image of a representative embryo co-expressing CIBN::pmGFP and mCherry::CRY2-OCRL 10 min after photo-activation, achieved as described in (C). mCherry::CRY2-OCRL is shown. The red box indicates the photo-activated area, which corresponds to (D’). In (D’), CIBN::pmGFP is shown. (E) Mean levels of mCherry::CRY2-OCRL plasma membrane recruitment with respect to the photo-activated area. The x coordinates represent cell IDs, with 0 indicating cells that are completely included in the photo-activation box (yellow cells in D and D’); −1 and 1 indicating cells that are partially included in the photo-activation box (green and blue cells in D and D’, respectively); −2, −3, −10 and 2, 3, 10 indicating, respectively, cells that are two, three, and ten cells away from cell 0 on either side of the photo-activation area. The y coordinates represent the log_2_ ratio of mCherry::CRY2-OCRL plasma membrane to cytosol fluorescence intensities. Pooled data are represented as mean ± SD (n = 3 embryos for each condition). (F–I) Still frames from a confocal movie of the ventral mesoderm of a representative embryo co-expressing CIBN::pmGFP and mCherry::CRY2-OCRL before photo-activation (F) and 10 min (G) and 20 min (H) after the beginning of two-photon illumination. The red box in (F) indicates the photo-activated area. Photo-activation was started before the beginning of ventral furrow formation and was achieved with 950-nm laser light, for a total scanning time of 2.5 s every 30 s. (I) Confocal image of the same embryo 30 min after the beginning of photo-activation. CIBN::pmGFP is shown. Scale bars, 10 μm. See also Figures S3, Figure S4, and Movie S4.

In order to control apical constriction with spatial precision, we used two-photon illumination (Denk et al., 1990). In fact, the requirement for simultaneous absorption of two photons means that photo-activation can be localized to the area where the laser is focused. We screened a range of wavelengths to set up the conditions for optimal photo-activation. 950 nm (475 nm × 2) was the most effective wavelength in triggering the translocation of CRY2-OCRL from the cytosol to the plasma membrane. Illumination at 900 nm (450 nm × 2) also resulted in CRY2-OCRL recruitment to the plasma membrane, albeit with slightly slower kinetics, while 1,000-nm illumination resulted only in minimal activation (Figure 4A).

However, two-photon illumination was less effective in activating CRY2-OCRL recruitment to the plasma membrane when compared to one-photon activation. The ratio of CRY2-OCRL plasma membrane versus cytosol fluorescence was 2 with 950-nm light and ∼3 with 488-nm light (Figure 4A). Therefore, in order to achieve higher levels of photo-activation with two-photon illumination, we used a different imaging protocol. Instead of a single z stack, five consecutive z stacks (separated by 1 μm) were illuminated for ∼500 ms each, with a total scan time of 2.5 s followed by a 30-s interval, during which embryos were illuminated at 561 nm to visualize CRY2-OCRL. The implementation of this protocol allowed us to achieve the same levels of activation with two-photon as with one-photon illumination, although with slower kinetics (Figure 4B). The t_1/2_ (calculated on the time of exposure at 950 nm or 488 nm) was ∼9 s at 950 nm, compared to 1 s at 488 nm. The association of CRY2-OCRL with CIBN anchored at the plasma membrane was reversible (Figure S3A), with kinetics similar to those obtained using one-photon activation (t_1/2_ of ∼9.2 min at room temperature). The two-photon laser power used for triggering CRY2-OCRL translocation to the plasma membrane was equivalent to the one normally used for EGFP imaging (3.0 mW measured at 1 cm from the objective) and did not result in any cytotoxic effect on the embryo or defects in ventral furrow formation, which proceeded with normal dynamics (Figures S3B–S3D). Furthermore, we could show that translocation of CRY2-OCRL to the plasma membrane correlated with the laser power that was used to trigger photo-activation (Figure 4C). Consistently, the average number of apically constricting cells during ventral furrow formation was a decreasing function of the laser power applied to the samples, with more cells undergoing apical constrictions when activated using a lower laser power (Figures S3E–S3P). These results show that, by increasing or decreasing the laser power used to trigger CRY2-OCRL translocation to the plasma membrane, it is possible to modulate the extent to which apical constriction is inhibited.

One of the major advantages of two-photon microscopy is that it can be used to image thick samples, as longer wavelengths have higher penetration depth than shorter wavelengths. Therefore, we tested whether this optogenetic approach could be used also in thicker specimens. We triggered the translocation of CRY2-OCRL to the plasma membrane at different depths from the apical surface. Figure S4A shows that photo-activation can be achieved at optimal levels until 80 μm deep inside the embryo.

Finally, we checked whether two-photon illumination would be an effective way to obtain precise spatial activation. We triggered local CRY2-OCRL plasma membrane recruitment in an area of known dimensions. After 10 min of continuous two-photon illumination, we measured the levels of CRY2-OCRL plasma membrane recruitment in both the illuminated area and the non-illuminated area. The quantification of photo-activation levels shows that CRY2-OCRL plasma membrane recruitment remained limited to the illuminated area. In cases where the illuminated area included only half of a cell, the pool of CRY2-OCRL recruited at the plasma membrane diffused only to the other half of the cell (Figures 4D–4E).

These results demonstrate that this two-photon illumination protocol allows precise spatial photo-activation with cellular resolution.

### Local Inhibition of Cell Contractility in a Subgroup of Ventral Cells Causes Arrest of Ventral Furrow Formation and Coordinated Contractions

Having established conditions for local photo-activation, we next tested the degree to which apical constriction in ventral cells contributes to ventral furrow formation. To directly address this point, we triggered activation of CRY2-OCRL recruitment to the plasma membrane only in ventral cells either at the onset of apical constriction or after cells were already constricted and tissue bending had initiated. These treatments resulted in the inhibition of tissue invagination (Figures 4F–4I; Movie S4) and in the reversion of the invagination process when the pulse of light was given after tissue bending had already started (Figures S4B–S4E). Together, these results demonstrate that apical constrictions in ventral cells are required throughout the invagination process.

**Figure 5:**
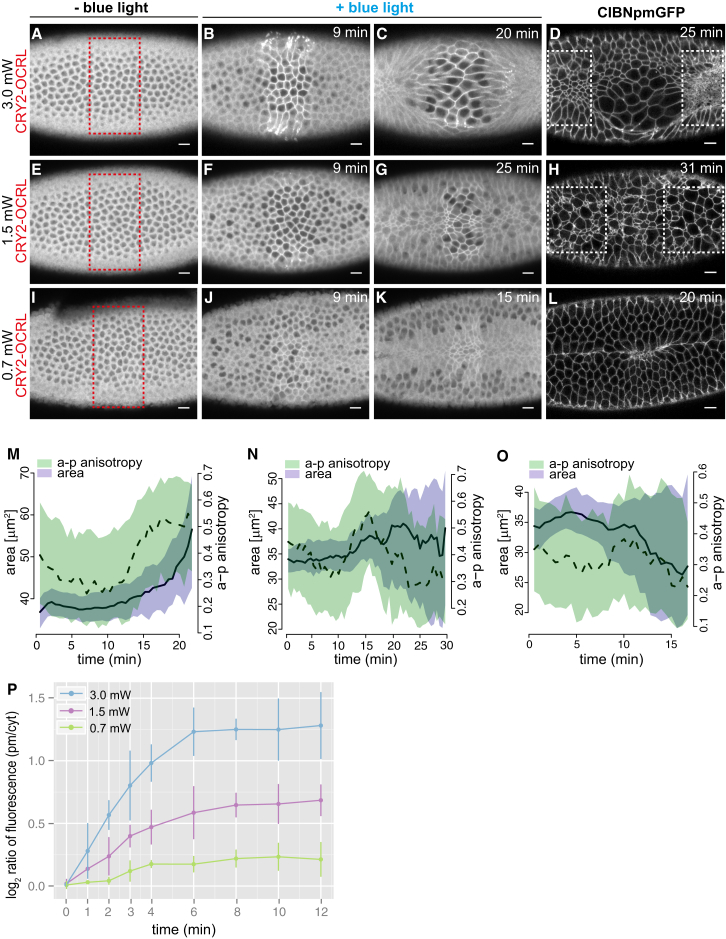
Local Inhibition of Apical Constriction in a Subgroup of Ventral Cells Causes Arrest of Ventral Furrow Formation and Coordinated Contractions (A–C) Still frames from a confocal movie of the ventral mesoderm of a representative embryo co-expressing CIBN::pmGFP and mCherry::CRY2-OCRL before photo-activation (A) and at 9 min (B) and 20 min (C) after photo-activation. The red box in (A) indicates the photo-activated area. Photo-activation was achieved with 950-nm laser light, for a total scanning time of 2.5 s every 30 s (laser power = 3.0 mW). (D) Confocal image of the same embryo 25 min after the first pulse of light. CIBN::pmGFP is shown. Cells in non-activated areas (white boxes) are hyperconstricted (n = 5 embryos). (E–G) Still frames from a confocal movie of the ventral mesoderm of a representative embryo co-expressing CIBN::pmGFP and mCherry::CRY2-OCRL before photo-activation (E) and at 9 min (F) and 25 min (G) after photo-activation. The red box in (E) indicates the photo-activated area. Photo-activation was achieved with 950-nm laser light, for a total scanning time of 2.5 s every 30 s (laser power = 1.5 mW). (H) Confocal image of the same embryo 31 min after the first pulse of light. CIBN::pmGFP is shown. Differently from (D), some of the cells contained within the photo-activated area are constricted, while some other cells are not. Notably, neighboring non-activated cells (white boxes) display a similar pattern of contractility to photo-activated cells (n = 3 embryos). (I–K) Still frames from a confocal movie of the ventral mesoderm of a representative embryo co-expressing CIBN::pmGFP and mCherry::CRY2-OCRL before photo-activation (I) and at 9 min (J) and 15 min (K) after photo-activation. The red box in (I) indicates the photo-activated area. Photo-activation was achieved with 950-nm laser light, for a total scanning time of 2.5 s every 30 s (laser power = 0.7 mW). (L) Confocal image of the same embryo 20 min after the first pulse of light. CIBN::pmGFP is shown. Both photo-activated cells and neighboring non-activated cells constricted and ventral furrow formed (n = 3 embryos). (M–O) Quantification of cell area (purple) and a-p anisotropy (green) for the photo-activated cells in (A–C, red box) (M), for the photo-activated cells in (E–G, red box) (N), and for the photo-activated cells in (I–K, red box) (O). The y coordinates represent cell area expressed in squared microns (left y axis) and a-p anisotropy (right y axis). The x axis represents time in minutes. Solid and dashed lines indicate the median over all cells for cell area (solid) and a-p anisotropy (dashed). Shaded regions show the interquartile range. (P) Mean levels of mCherry::CRY2-OCRL recruitment to the plasma membrane in response to different laser powers (3.0 mW, 1.5 mW, and 0.7 mW). The y axis shows the log_2_ ratio of the membrane to cytoplasmic fluorescence intensity of mCherry::CRY2-OCRL in photo-activated cells. The x axis displays time in min, with 0 corresponding to the beginning of photo-activation. Pooled data are represented as mean ± SD (n ≥ 3 embryos for each condition). Scale bars, 10 μm. See also Movies S5A, S5B, and S5C.

Next, we tested whether the tunable feature of CRY2-OCRL plasma membrane recruitment at different laser powers (Figure 4C) could be used to differentially modulate apical constriction in only a subgroup of cells located in the middle of the ventral tissue and, thus, allow us to probe the extent of force integration and cell-cell coordination necessary to drive invagination. We tested three different laser powers: 3.0, 1.5, and 0.7 mW. When the laser power was set to 3.0 mW, which corresponded to the power used in all previous experiments and for which we measured optimal levels of activation (see Figure 4C), cells within the activated area failed to constrict and elongated along the embryo a-p axis, suggesting that pulling forces by non-activated neighboring cells caused them to stretch (Figures 5A–5C and 5M; Movie S5A). Consistently, in toto imaging after activation revealed that cells outside of the photo-activated area were hyper-constricted (Figure 5D). Using a lower laser power (1.5 mW), when the system was activated at sub-optimal levels, some cells within the activated areas retained the capability to contract and did not elongate along the a-p axis (Figures 5E–5G and 5N; Movie S5B). This phenotype correlated with cessation of contractility in non-activated cells (Figure 5H) and also resulted in a global arrest of ventral furrow invagination. Lastly, at a lower laser power (0.7 mW), almost all the cells contained in the activated area retained the capability to contract and eventually invaginate, albeit with lower levels of a-p anisotropy when compared to WT embryos (Figures 5I–5L and 5O; Movie S5C). Together, these data reveal highly coordinated contractile behavior during tissue invagination and uncover a dual response of non-activated cells that correlates with the levels of optogenetic activation (Figure 5P) and therefore, presumably, with different degrees of changes in tissue tension.

**Figure 6:**
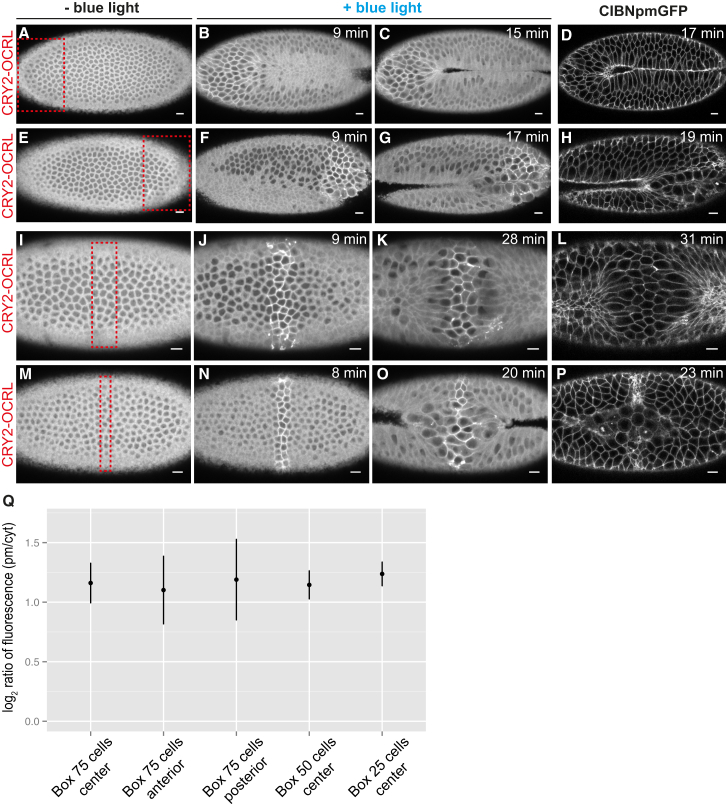
The Number of Cells in which Apical Constriction Is Inhibited Influences the Capability of Neighboring Cells to Form a Furrow (A–C) Still frames from a confocal movie of the ventral mesoderm of a representative embryo co-expressing CIBN::pmGFP and mCherry::CRY2-OCRL before photo-activation (A) and at 9 min (B) and 15 min (C) after the beginning of photo-activation. The red box in (A) indicates the photo-activated area, which partially overlaps, in the a-p direction, the presumptive mesoderm. Photo-activation was achieved with 950-nm laser light, for a total scanning time of 2.5 s every 30 s (laser power = 3.0 mW). (D) Confocal image of the same embryo taken 17 min after the first pulse of light. CIBN::pmGFP is shown (n = 3 embryos). (E–G) Still frames from a confocal movie of the ventral mesoderm of a representative embryo co-expressing CIBN::pmGFP and mCherry::CRY2-OCRL before photo-activation (E) and at 9 min (F) and 17 min (G) after the first pulse of local photo-activation. The red box in (E) indicates the photo-activated area, which partially overlaps, in the a-p direction, the presumptive mesoderm. Photo-activation was achieved with 950-nm laser light, for a total scanning time of 2.5 s every 30 s (laser power = 3.0 mW). (H) Confocal image of the same embryo taken 19 min after the first pulse of light. CIBN::pmGFP is shown (n = 3 embryos). (I–K) Still frames from a confocal movie of the ventral mesoderm of a representative embryo co-expressing CIBN::pmGFP and mCherry::CRY2-OCRL before photo-activation (I) and at 9 min (J) and 28 min (K) after the beginning of photo-activation. The red box in (I) indicates the photo-activated area. Photo-activation was achieved with 950-nm laser light, for a total scanning time of 2.5 s every 30 s (laser power = 3.0 mW). (L) Confocal image of the same embryo taken 31 min after the first pulse of light. CIBN::pmGFP is shown (n = 3 embryos). (M–O) Still frames from a confocal movie of the ventral mesoderm of a representative embryo co-expressing CIBN::pmGFP and mCherry::CRY2-OCRL before photo-activation (M) and at 8 min (N) and 20 min (O) after the beginning of photo-activation. The red box in (M) indicates the photo-activated area. Photo-activation was achieved with 950-nm laser light, for a total scanning time of 2.5 s every 30 s (laser power = 3.0 mW). (P) Confocal image of the same embryo taken 23 min after the beginning of photo-activation. CIBN::pmGFP is shown (n = 3 embryos). (Q) Comparison of mean photo-activation levels among embryos where photo-activation was achieved in boxes of different sizes and positions within the furrow primordium. The x coordinates represent samples. The y coordinates represent the log_2_ ratio of CRY2-OCRL plasma membrane (pm) to cytosol (cyt) fluorescence intensity. Pooled data are represented as mean ± SD (n ≥ 3 embryos for each condition). Scale bars, 10 μm. See also Movies S6A and S6B and Movies S7A and S7B.

The aforementioned results illustrate that inhibition of contractility in a subgroup of ∼75 cells located in the middle of the ventral furrow resulted in a global arrest of ventral furrow formation. Next, we asked whether this inhibitory effect depended on the position where photo-activation was performed along the a-p axis or rather on the number of cells that were inhibited in their contractile behavior. Using the laser power that triggered maximal levels of activation (3.0 mW), we prevented contractility in a group of 75 cells in an eccentric position toward the anterior or posterior end of the primordium. Under this condition, ventral furrow formed at the non-activated side of the tissue (Figures 6A–6H; Movies S6A and S6B), suggesting that, if enough cells along the furrow primordium are free to constrict, they can generate the force necessary for invagination. To further test this hypothesis, we narrowed the area of photo-activation to two incrementally smaller subgroups of cells located in the middle of the ventral furrow tissue. When CRY2-OCRL plasma membrane recruitment was triggered in a subgroup of ∼50 cells, none of the embryos formed a complete furrow, and in toto imaging after activation showed that cells located in the neighboring non-activated areas were hyper-constricted and did not invaginate or formed only a shallow indentation (Figures 6I–6L; Movie S7A). However, when CRY2-OCRL plasma membrane recruitment was triggered in only a subgroup of ∼25 cells, non-activated cells could invaginate, forming two small furrows on either side of the activated area (Figures 6M–6P; Movie S7B). Importantly, all photo-activation resulted in equal amounts of CRY2-OCRL plasma membrane recruitment (Figure 6Q). Together, these results suggest that, if enough cells are free to constrict, they can locally coordinate and build sufficient tension to drive invagination.

### Anisotropy of Cell Shape Changes within Individual Cells Depends on the Geometry of the Contractile Ventral Furrow Primordium

**Figure 7:**
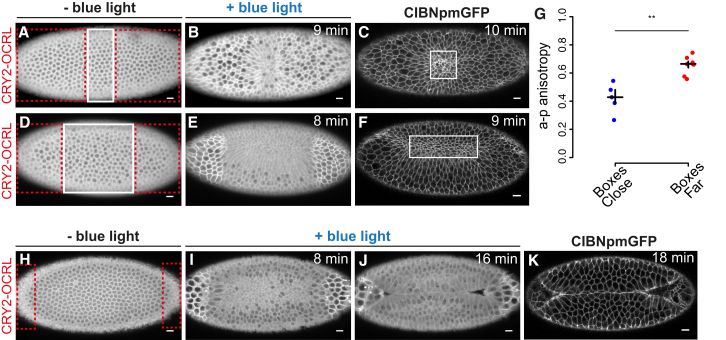
The Geometry of the Ventral Furrow Primordium Determines the Degree of Anisotropy of Cell Shape Changes in Individual Cells (A and B) Still frames from a confocal movie of the ventral mesoderm of a representative embryo co-expressing CIBN::pmGFP and mCherry::CRY2-OCRL before photo-activation (A) and 9 min after the beginning of photo-activation (B). Red boxes in (A) indicate photo-activated areas, and the white box indicates a non-activated area where cells can constrict. Photo-activation was achieved by illuminating each area with 950-nm laser light, for a total scanning time of 2.5 s every 30 s. (C) Confocal image of the same embryo taken 10 min after the beginning of photo-activation. CIBN::pmGFP is shown. Anisotropy along the embryo a-p axis was calculated only for constricting cells (white box) in the non-activated area (n = 5 embryos). (D and E) Still frames from a confocal movie of the ventral mesoderm of a representative embryo co-expressing CIBN::pmGFP and mCherry::CRY2-OCRL before photo-activation (D) and 8 min after the beginning of photo-activation (E). Red boxes in (D) indicate photo-activated areas, and the white box indicates a non-activated area where cells can constrict. Photo-activation was achieved as described in (B). (F) Confocal image of the same embryo taken 9 min after the first pulse of light. Anisotropy along the embryo a-p axis was calculated only for constricting cells (white box) in the non-activated area (n = 8 embryos). (G) Comparison of median a-p anisotropy of non-activated constricting cells between samples in dependence of the distance between the photo-activated areas. Values along the y axis represent a-p anisotropy. The degree of a-p anisotropy is higher when the boxes are placed farther apart than when they are closer together. Each dot represents a single embryo. The crosses show group median and interquartile range. p = 4.3 × 10^−3^, two-sample Welch’s t test. (H–J) Still frames from a confocal movie of the ventral mesoderm of a representative embryo co-expressing CIBN::pmGFP and mCherry::CRY2-OCRL before photo-activation (H) and at 8 min (I) and 16 min (J) after the beginning of photo-activation. The red boxes in (H) indicate photo-activated areas. Photo-activation was achieved as described in (B). (K) Confocal image of the same embryo taken 18 min after the first pulse of light (n = 3 embryos). Scale bars, 10 μm. See also Figure S5 and Movies S8A, S8B, and S9.

Current models suggest that a-p anisotropic constriction in ventral cells results from cell-cell interaction among neighboring cells that pull on each other (Martin et al., 2010). According to this proposal, a-p anisotropy might be a consequence of the rectangular geometry of the ventral furrow tissue. Indeed, the number of cells that undergo constriction is higher along the a-p axis than the dorsal-ventral (d-v) axis. This might cause a higher tissue tension along the a-p axis, which, in turn, will force cells to constrict preferentially along the d-v axis and elongate along the a-p axis. In order to test this model, we asked whether reducing the number of contractile cells along the a-p axis would cause any change in the degree of a-p anisotropic constriction. Using two-photon laser illumination, we triggered CRY2-OCRL plasma membrane recruitment in two groups of cells: one at the posterior end and one at the anterior end of the ventral furrow tissue (Figures 7A–7F; Movies S8A and S8B). As shown in Figure 7G, the degree of a-p anisotropy in non-activated cells was higher if the two areas of photo-activation were further apart (when the rectangular geometry of non-activated tissue was preserved) than if they were closer together (non-activated tissue of oblong geometry perpendicular to the a-p axis). This result shows that the rectangular geometry of the ventral furrow tissue plays an important role in determining the emergence of asymmetric constriction. However, as in neither of the aforementioned perturbations ventral furrow invagination occurred (Figures S5A–S5H), our results also suggest that a-p anisotropy per se is not sufficient to predict whether tissue invagination will occur. Importantly, inhibition of cell constriction in two smaller boxes (containing ∼25 cells each) at the anterior and posterior regions of the ventral furrow primordium did not block ventral furrow formation (Figures 7H–7K; Movie S9), which further suggests that, if enough cells are able to collectively constrict, they can generate the force necessary for tissue invagination.

Taken together, these results support previous genetic analyses showing that the machinery controlling the contractility of ventral cells is not polarized along the a-p axis (Mason et al., 2013) and suggest that the rectangular geometry of the ventral furrow tissue plays an important role in determining the emergence of asymmetric constriction and contractile group behavior.

## Discussion

Optogenetics is a powerful technique that allows the control of protein activity with light. Its application to modulate cell-biological processes in vivo, especially during morphogenesis of multicellular organisms, might thus provide an effective tool to regulate developmental processes with high spatiotemporal precision. In this report, we developed an optogenetic method that allows modulation of cell contractility during tissue morphogenesis, and we demonstrate the validity of this approach in addressing the interplay between tissue geometry and force transmission during ventral furrow formation.

The data collected demonstrate that depletion of PI(4,5)P_2_ from the plasma membrane causes loss of apical constriction during ventral furrow formation. Consistent with the importance of PI(4,5)P_2_ in cortical actin recruitment (Bardet et al., 2013, Comer and Parent, 2007, Reversi et al., 2014), we observed a clear loss of actin (as revealed by Moesin) at the apical surface upon optogenetic activation. It is likely that the effects we observed are caused by the loss of multiple actin regulators. Possible candidates include components of the Rho pathway, which have been shown previously to be required for apical constriction in ventral cells (Barrett et al., 1997, Fox and Peifer, 2007, Kölsch et al., 2007, Mason et al., 2013).

The optogenetic system that we used is based on the CRY2-CIB1 protein dimerization module, which is particularly suitable for application in living organisms, as it can be rapidly activated by blue-light illumination, and it does not require the addition of an exogenous chromophore. This is particularly relevant in the case of embryos that develop inside non-permeable layers (e.g., the *Drosophila* embryo) that would otherwise require the microinjection of exogenous molecules. One drawback of the CRY2-CIB1 module when compared to other light dimerization systems, such as the Phytochrome B (PHYB)/Phytochrome-interacting factor (PIF) module (Levskaya et al., 2009), is that the interaction between CRY2 and CIB1 cannot be reverted in a regulated manner using light. For example, the possibility to precisely activate and de-activate the interaction between PHYB and PIF using red and infrared light, respectively, has been exploited to generate localized patterns of activation in cell culture (Toettcher et al., 2013). However, the PHYB/PIF system requires the addition of an exogenous chromophore.

To circumvent the limitation associated with the CRY2/CIB1 module, we developed a two-photon-based photo-activation protocol that, by limiting light scattering, allows the generation of precise patterns of activation with cellular precision (see Figures 4D and 4E). However, activation in two-photon illumination is slower than in one-photon illumination (1 s versus 9 s), and, when combined with one-photon imaging, it requires an additional time lag of 30 s to switch between the two imaging modes. Therefore, in order to control morphogenetic processes that occur with fast dynamics (<5 min), it could be advantageous to use a non-tagged CIBN plasma membrane anchor and GFP reporters of interest. This approach would allow simultaneous photo-activation and imaging; thus, it would improve the time resolution and also prevent the reversibility of optogenetic activation. The use of a single-wavelength excitation, dual-color, two-photon acquisition probe such as mKeima (Kawano et al., 2008), in combination with EGFP, could also offer a valid alternative.

The results (Figures 4F–4I; Figures S4B–S4E; Movie S4) illustrate that apical constriction is not only required to initiate tissue invagination but also required throughout the invagination process. These data are consistent with previous studies suggesting that a supracellular contractile actomyosin meshwork in ventral cells is required to generate a tissue-level force that drives invagination (Martin et al., 2009, Martin et al., 2010). We interpret the loss of coordinated contractile behavior upon local inhibition of cell contractility in the middle of the ventral furrow tissue as likely being a consequence of interfering with long-range force transmission along the supracellular actomyosin meshwork. Interestingly, modulation of apical constriction at different laser powers reveals a dual contractile response of non-activated cells to the level of optogenetic activation. Higher levels of optogenetic activation cause activated cells to become elongated along the a-p axis, suggesting that they are being pulled by non-activated cells, which are indeed hyper-constricted (Figures 5A–5D; Movie S5A). At lower photo-activation levels, cells in the activated region display a resistance to deformation along the a-p axis, and cells in the non-activated regions fail to constrict (Figures 5E–5H; Movie S5B). Different contractile responses in non-activated cells might simply reflect a change in the overall balance of forces produced and experienced by these cells. Alternatively, assuming that the level of optogenetic activation correlates with a corresponding reduction in tissue tension, these results might suggest that cells respond to changes in tissue tension either by hyper-contracting, when tension drops below a certain threshold, or by reducing contractility, when tension remains higher than this threshold. Both types of responses, taken individually in different contexts, have been described previously. During dorsal closure, laser-cut experiments demonstrated that lowering tissue tension results in the arrest of cell contractility not only in cells immediately next to the cut but also in cells further away (Solon et al., 2009). This observation suggests that stretching from neighboring contracting cells is required to initiate cell constrictions. On the other hand, during ventral furrow formation, reducing cell adhesion caused cells next to epithelial tears to constrict isotropically (Martin et al., 2010), which suggests that lowering tissue tension facilitates cell-autonomous constrictions (Mason et al., 2013). Interestingly, a recent report demonstrates that, during ventral furrow formation, cells that are stabilized in their shape after contracting have a higher probability of having neighboring contractions (Xie and Martin, 2015). Future experiments are needed to test whether, during ventral furrow formation, mechano-sensitive signaling systems exist to buffer variation in contractile efficiency among individual cells, in order to facilitate coordinated collective contractions and to test whether other actomyosin-dependent morphogenetic processes are regulated in a similar manner.

Given the conserved functions of apical constriction during many morphogenetic movements, including tube formation and neurulation, the application of this optogenetic approach should greatly facilitate our understanding of tissue mechanics during animal development.

## Experimental Procedures

### Live Imaging and Photo-activation

Embryos were staged and mounted in a dark room using a standard upright microscope in which the light source was replaced with a yellow-light lamp to avoid unwanted photo-activation. Embryos at the right developmental stage were imaged with a Zeiss LSM 780 NLO confocal microscope (Carl Zeiss) using a C-Apochromat 63×/NA 1.2 water immersion objective (Carl Zeiss).

Photo-activation with one-photon illumination was achieved using a continuous-wave argon laser (λ = 488 nm) by illuminating one z stack 5 μm from the apical surface, for a total scan time of 1 s. Between two consecutive excitations, there was an interval of 30 s and the acquisition of the mCherry channel at 561 nm. The laser power used was equivalent to the one used for EGFP imaging (6.9 μW, measured 1 cm from the objective).

Photo-activation with two-photon illumination was achieved using a femtosecond (140-fs) pulsed laser (Chameleon Ultra II; Coherent) at a repetition rate of 80 MHz, with λ = 950 nm by illuminating five consecutive z stacks (each plane separated by 1 μm) for ∼500 ms each at a scanning speed of 1 μs/pixel, using a 2× frame averaging setting in bidirectional mode, for a total scan time of 2.5 s. Unless stated otherwise, the laser power used was 3.0 mW (measured 1 cm from the objective), equivalent to the one normally used for imaging of EGFP. For local photo-activations, the first frame was acquired at 561 nm to image CRY2-OCRL prior to activation. Between two consecutive two-photon excitations, there was an interval of 30 s and the acquisition of the mCherry channel at 561 nm. Further details are available in Supplemental Experimental Procedures.

### Image Analysis

Images were processed using custom-written R software that is available as part of the experiment data package furrowSeg (http://www.bioconductor.org). In brief, cell membrane was identified using Gaussian smoothing (filter size, ∼1 μm), adaptive thresholding, and object filtering. Masks representing cytoplasmic regions were then input into a Voronoi-based segmentation algorithm that accurately identified cell boundaries. Cell area and a-p anisotropy were calculated from the resulting object masks for each z stack separately.

The level of optogenetic activation of a cell was calculated by manually defining the membrane mask with the EGFP channel and removing the nuclei, which tended to be darker than true cytoplasmic regions. Then, the level of optogenetic activation of each cell was quantified by taking the log_2_ ratio of the average membrane to average cytosol intensities. The same procedure was used to quantify the plasma membrane levels of PH_PLCδ_::GFP and Moesin::mCherry. Further details are described in the Supplemental Experimental Procedures.

### Statistics and Data Analysis

A table of cell features extracted from the image processing is freely available as part of the experiment data package furrowSeg (http://www.bioconductor.org), along with descriptions and runnable code that automatically reproduces the statistics and data analysis performed for this article. In brief, we used a combination of manual and automated methods to identify regions of interest in the images. Testing for differences between median cell area and a-p anisotropy across conditions was done using two-sided, two-sample Student’s or Welch's t tests, as specified in figure legends. Where appropriate, multiple testing correction was performed using the method of Bonferroni.

### Cloning and Fly Genetics

Cloning and fly genetics details are described in the Supplemental Experimental Procedures.

## Author Contributions

The research was conceived and designed by G.G., J.D.B., and S.D.R. G.G. performed the experiments. G.G., J.D.B., and S.D.R. analyzed the data with the help of W.H.; G.G. and S.D.R. wrote the manuscript.

## Supplemental Information

Document S1. Supplemental Experimental Procedures and Figures S1–S5

Movie S1. Ventral Furrow Formation Dynamics in a Control Embryo, Related to Figure 2

Movie S2. The Effects of Global Activation of CRY2-OCRL Recruitment to the Plasma Membrane on Ventral Furrow Formation, Related to Figure 2

Movie S3. Ventral Furrow Formation Progresses Normally in Non-activated *sktl*^*+/−*^ and *zip*^*+/−*^ Heterozygous Embryos, Related to Figure 3

Movie S4. Local Activation of mCherry::CRY2-OCRL Recruitment to the Plasma Membrane Results in the Inhibition of Ventral Furrow Formation, Related to Figure 4

Movie S5. Modulation of Apical Constriction at Different Laser Powers, Related to Figure 5

Movie S6. Local Inhibition of Apical Constriction at the Anterior or Posterior Ends of the Ventral Furrow Tissue, Related to Figure 6

Movie S7. Local Inhibition of Apical Constriction in the Middle of the Ventral Furrow Tissue, Related to Figure 6

Movie S8. The Impact of Tissue Geometry on Contractile Behavior, Related to Figure 7

Movie S9. Ventral Furrow Formation Depends on the Number of Cells that Can Constrict, Related to Figure 7

Document S2. Article plus Supplemental Information
