# Supplementary material for: An Optogenetic Method to Modulate Cell Contractility during Tissue Morphogenesis
Source: Dev Cell. 2015 Dec 7;35(5):646–60. doi: 10.1016/j.devcel.2015.10.020 (PMC4683098; doi:10.1016/j.devcel.2015.10.020)
Supplement: Document S1. Supplemental Experimental Procedures and Figures S1–S5 [file mmc1.pdf]

Developmental Cell

Supplemental Information

An Optogenetic Method to Modulate Cell Contractility during Tissue Morphogenesis

Giorgia Guglielmi, Joseph D. Barry, Wolfgang Huber, and Stefano De Renzis

Supplemental Figures

Figure S1

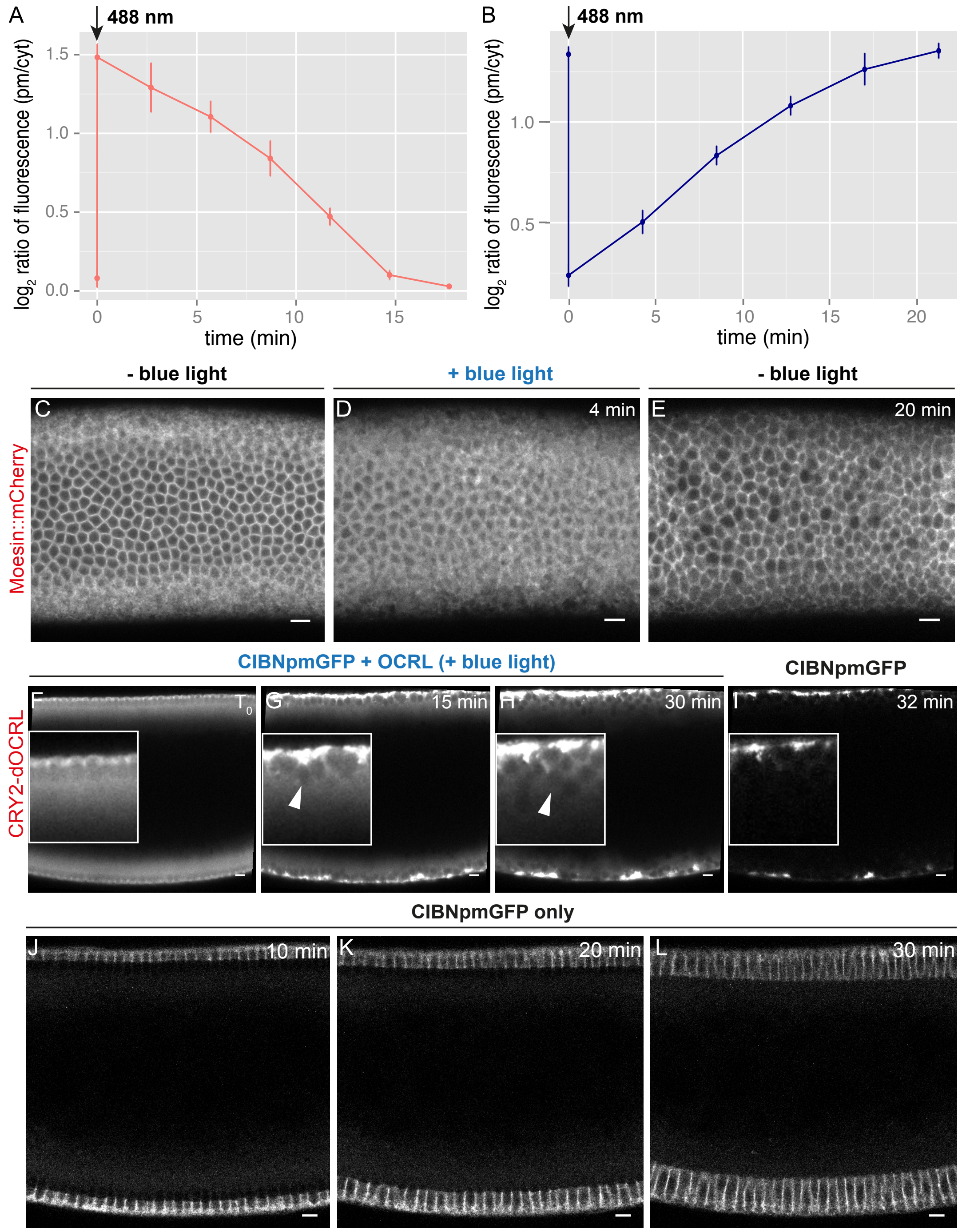

Figure S2

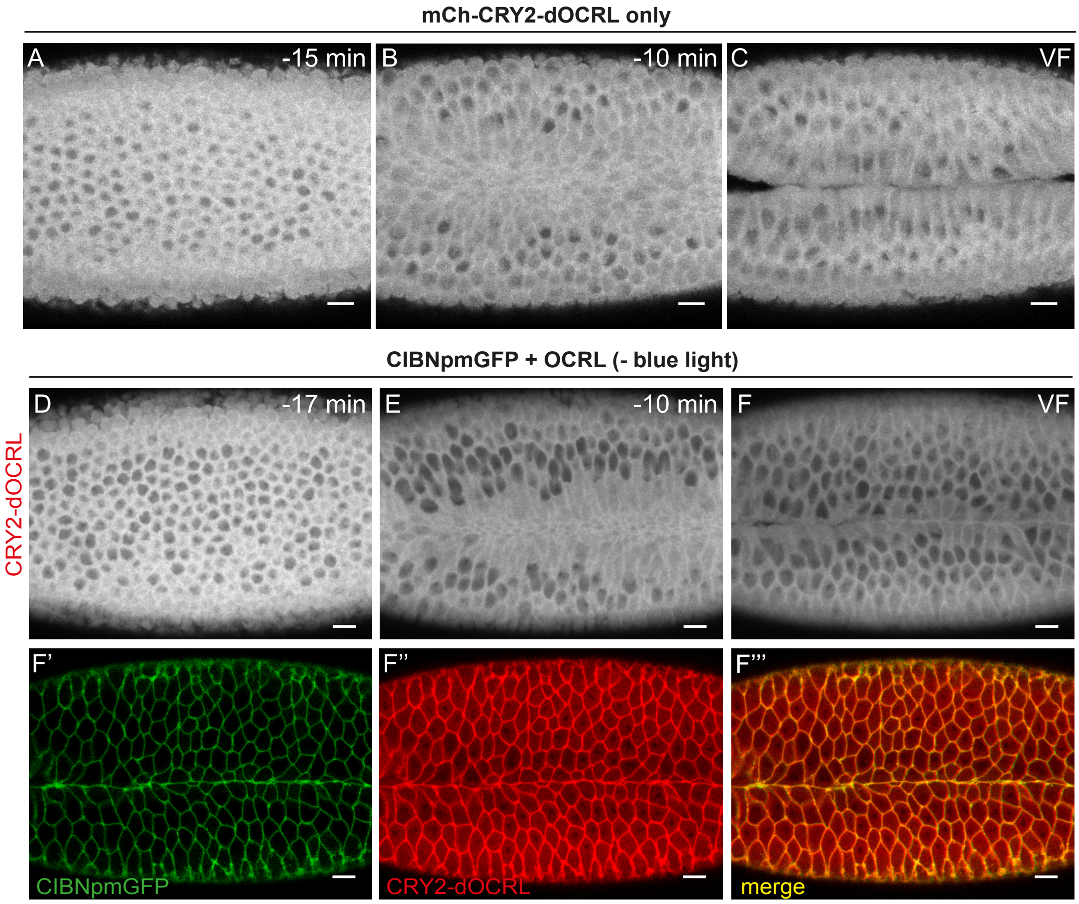
mCh-CRY2-dOCRL only
A
-15 min
B
-10 min
C
VF
CIBNpmGFP + OCRL (- blue light)
CRY2-dOCRL
D
-17 min
E
-10 min
F
VF
F'
F''
F'''
CIBNpmGFP
CRY2-dOCRL
merge

Figure S3

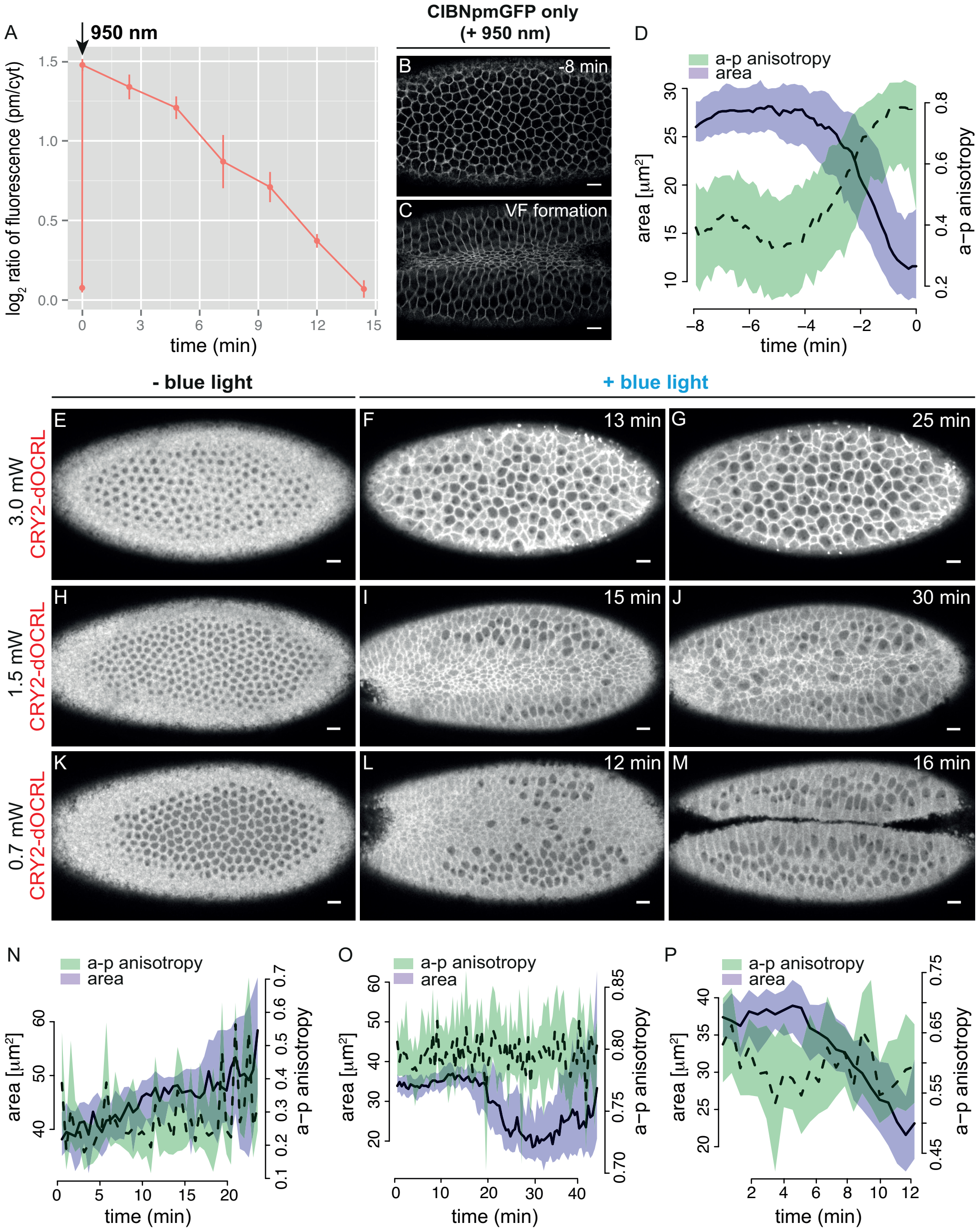
A
950 nm
log2 ratio of fluorescence (pm/cyt)
time (min)
CIBNpmGFP only
(+ 950 nm)
B
-8 min
C
VF formation
D
a-p anisotropy
area
area [µm²]
a−p anisotropy
time (min)
- blue light
+ blue light
E
F
13 min
G
25 min
3.0 mW
CRY2-dOCRL
H
I
15 min
J
30 min
1.5 mW
CRY2-dOCRL
K
L
12 min
M
16 min
0.7 mW
CRY2-dOCRL
N
O
P
a-p anisotropy
area
area [µm²]
a−p anisotropy
time (min)

Figure S4

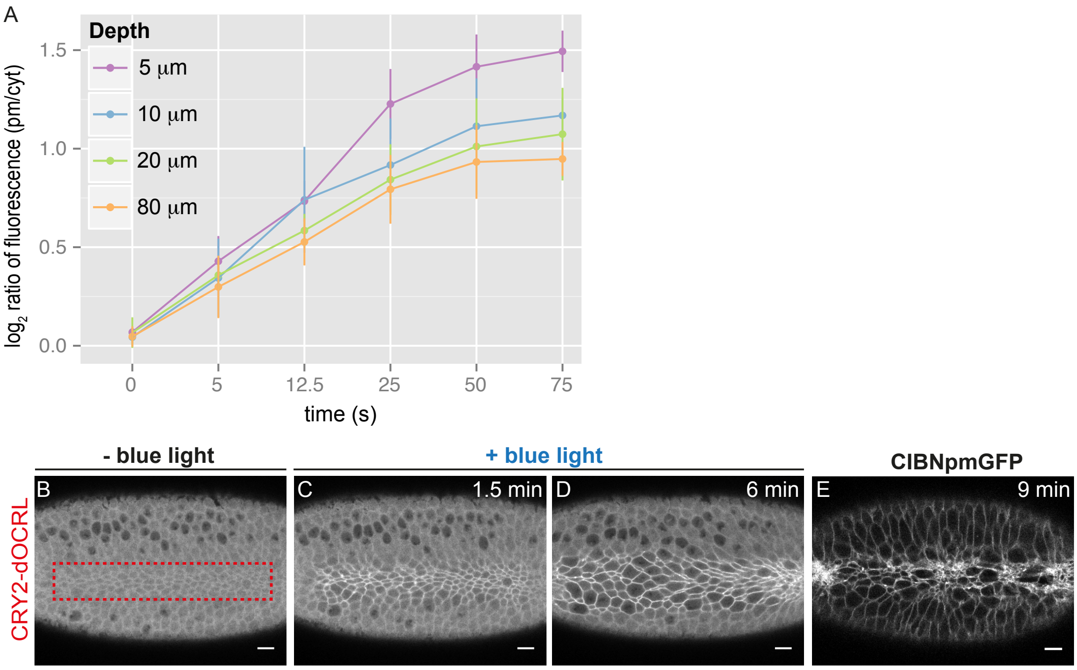
A
Depth
5 μm
10 μm
20 μm
80 μm
log2 ratio of fluorescence (pm/cyt)
1.5
1.0
0.5
0.0
0
5
12.5
25
50
75
time (s)
- blue light
+ blue light
CIBNpmGFP
B
C
1.5 min
D
6 min
E
9 min
CRY2-dOCRL

Figure S5

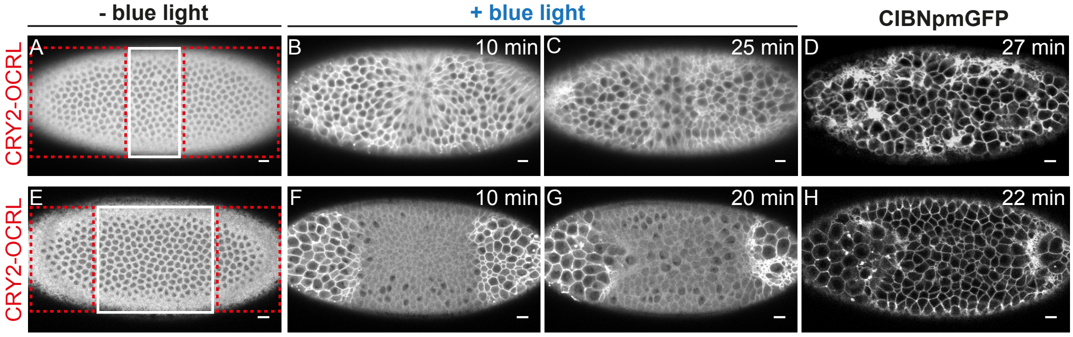

- blue light
+ blue light
CIBNpmGFP
CRY2-OCRL
CRY2-OCRL
A
B
10 min
C
25 min
D
27 min
E
F
10 min
G
20 min
H
22 min

Supplemental Figure Legends

Figure S1, related to Figure 1. CRY2-OCRL plasma membrane recruitment is reversible upon switching off blue light illumination.

(A) Mean levels of plasma membrane-associated mCherry::CRY2-OCRL in the dark, following photo-activation (black arrow). Photo-activation was achieved at a scanning speed of $1.27x10^{-6}$ s/pixel for a total time of 1s for the entire embryo at 30 s intervals for 4 min using a continuous wave laser (488 nm). x axis displays time (in min). y axis displays the log_2 ratio of mCherry::CRY2-OCRL plasma membrane to cytosol fluorescence intensities. Pooled data are represented as mean ± SD (n=3 embryos).

(B) Mean levels of cortical Moesin::mCherry levels in the dark, following photo-activation (black arrow). Photo-activation was achieved as in (A). x axis displays time (in min). y axis displays the log_2 ratio of Moesin::mCherry plasma membrane to cytosol fluorescence intensities. Pooled data are represented as mean ± SD (n=3 embryos).

(C-E) Confocal images of a representative embryo co-expressing a non-tagged version of mCherry::CRY2-OCRL, the actin-binding protein Moesin::mCherry and CIBN::pmGFP before photo-activation **(C)**, 4 min after the beginning of photo-activation **(D)**, and after 20 min in the absence of blue light **(E)**. Scale bars, 10 μm.

(F-I) Still frames from a time-lapse confocal movie of an optical cross-section of a representative embryo co-expressing CIBN::pmGFP and mCherry::CRY2-OCRL at the onset of photo-activation **(F)**, 15 min **(G)** and 30 min **(H)** after the beginning of photo-activation. Photo-activation was started at the beginning of cellularization. White arrows indicate the position of the nuclei, which are misaligned. **(I)** Confocal image of the same embryo taken 32 min after the first pulse of light. CIBN::pmGFP is shown. (n=5 embryos). Scale bars, 10 μm.

(J-L) Still frames from a time-lapse confocal movie of an optical cross-section of an embryo expressing only CIBN::pmGFP after 10 min **(J)**, 20 min **(K)** and 30 min **(L)** from the beginning of cellularization. Scale bars, 10 μm.

Figure S2, related to Figure 3. Embryos expressing either CRY2-OCRL only or co-expressing CRY2-OCRL and CIBN::pmGFP do not show developmental defects in the absence of blue light illumination.

(A-C) Still frames from a time-lapse confocal movie of the ventral mesoderm of a representative control embryo expressing only mCherry::CRY2-OCRL 15 min before **(A)**, 10 min before **(B)**, and at completion of ventral furrow (VF) invagination **(C)**. (n=3 embryos). Scale bars, 10 μm.

(D-F) Still frames from a time-lapse confocal movie of the ventral mesoderm of a representative embryo co-expressing CIBN::pmGFP and mCherry::CRY2-OCRL 17 min before **(D)**, 10 min before **(E)**, and at completion of ventral furrow (VF) invagination **(F)**. The embryo was imaged at 561 nm only. **(F')** shows CIBN::pmGFP , **(F'')** shows mCherry::CRY2-OCRL, and **(F''')** shows a merge of the two channels at completion of ventral furrow invagination. (n=5 embryos). Scale bars, 10 μm.

Figure S3, related to Figure 4. Inhibition of cell contractility correlates with the two-photon laser intensity used to trigger photo-activation.

(A) Mean levels of plasma membrane-associated mCherry::CRY2-OCRL in the dark, following photo-activation (black arrow). Photo-activation was achieved with a 950 nm laser light for a total scanning time of 2.5 s every 30 s for 10 min. x axis displays time (in min). y axis displays the log_2 ratio of mCherry::CRY2-OCRL plasma membrane to cytosol fluorescence intensities. Pooled data are represented as mean ± SD (n=3 embryos).

(B-C) Still frames from a confocal movie of the ventral mesoderm of a representative control embryo expressing only CIBN::pmGFP 8 min before ventral furrow formation **(B)** and at the onset of ventral furrow formation **(C)**. The embryo was continuously imaged with a 950 nm laser at a power of 3.0 mW. Scale bars, 10 μm.

(D) Quantification of cell area (purple) and anterior-posterior (a-p) anisotropy (green) for the control embryo shown in panels B-C. y-coordinates represent cell area expressed in squared microns (left y-axis) and a-p anisotropy (right y-axis). Values along the x-axis represent time in min. Solid and dashed lines indicate the median over all cells for cell area (solid) and a-p anisotropy (dashed). Shaded regions show the interquartile range. Time 0 corresponds to the time point of ventral furrow invagination.

(E-G) Still frames from a confocal movie of the ventral mesoderm of a representative embryo co-expressing CIBN::pmGFP and mCherry::CRY2-OCRL before photo-activation **(E)**, 13 min **(F)**, and 25 min **(G)** after the beginning of photo-activation, achieved as described in (A) (laser power = 3.0 mW). Scale bars, 10 μm.

(H-J) Still frames from a confocal movie of the ventral mesoderm of a representative embryo co-expressing CIBN::pmGFP and mCherry::CRY2-OCRL before photo-activation **(H)**, 15 min **(I)**, and 30 min **(J)** after photo-activation. Photo-activation was achieved as described in (A) (laser power = 1.5 mW). Scale bars, 10 µm.
(K-M) Still frames from a confocal movie of the ventral mesoderm of a representative embryo co-expressing CIBN::pmGFP and mCherry::CRY2-OCRL before photo-activation **(K)**, 12 min **(L)**, and 16 min **(M)** after the beginning of photo-activation. Photo-activation was achieved as described in (A) (laser power = 0.7 mW). Scale bars, 10 µm.
(N-P) Quantification of cell area (purple) and anterior-posterior (a-p) anisotropy (green) for the photo-activated embryo shown in panels E-G **(N)**, for the photo-activated embryo shown in panels H-J **(O)**, and for the photo-activated embryo shown in panels K-M **(P).** y-coordinates represent cell area expressed in squared microns (left y-axis) and a-p anisotropy (right y-axis). Values along the x-axis represent time in min. Solid and dashed lines indicate the median over all cells for cell area (solid) and a-p anisotropy (dashed). Shaded regions show the interquartile range.

Figure S4, related to Figure 4. Two-photon microscopy allows deep penetration into biological tissues and local inhibition of apical constriction.
(A) Mean levels of mCherry::CRY2-OCRL recruitment to the plasma membrane-anchored CIBN::pmGFP in response to 950 nm light at different depths (5 µm, 10 µm, 20 µm and 80 µm) inside the embryo. Photo-activation was achieved with a 950 nm laser light for a total scanning time of 2.5 s every 30 s. x-coordinates represent time of exposure to light (in s). y-coordinates represent the $\log_2$ ratio of mCherry::CRY2-OCRL plasma membrane to cytosol fluorescence intensities. Pooled data are represented as mean ± SD (n=3 embryos for each condition).
(B-E) Still frames from a confocal movie of the ventral mesoderm of a representative embryo co-expressing CIBN::pmGFP and mCherry::CRY2-OCRL before photo-activation **(B)**, 1.5 min **(C)** and 6 min **(D)** after the beginning of photo-activation. The red box in panel B indicates the photo-activated area. Photo-activation was achieved with a 2.5 s pulse of 950 nm light every 30 s. **(E)** Confocal image of the same embryo taken 9 min after the beginning of photo-activation. CIBN::pmGFP is shown. (n=3 embryos). Scale bars, 10 µm.

Figure S5, related to Figure 7. Blocking apical constriction in two groups of cells at both ends of the ventral furrow primordium results in the inhibition of tissue invagination.
(A-C) Still frames from a confocal movie of the ventral mesoderm of a representative embryo co-expressing CIBN::pmGFP and mCherry::CRY2-OCRL before photo-activation **(A)**, 10 min **(B)**, and 25 min **(C)** after the first pulse of light. Red boxes in panel A indicate photo-activated areas, the white box indicates non-activated area where cells can constrict. Photo-activation was achieved with a 2.5 s pulse of 950 nm light by illuminating each area every 30 s. **(D)** Confocal image of the same embryo taken 27 min after the first pulse of light. CIBN::pmGFP is shown. (n=3 embryos). Scale bars, 10 µm.
(E-G) Still frames from a confocal movie of the ventral mesoderm of a representative embryo co-expressing CIBN::pmGFP and mCherry::CRY2-OCRL before photo-activation **(E)**, 10 min **(F)**, and 20 min **(G)** after the first pulse of light. Red boxes in panel E indicate photo-activated areas, the white box indicates non-activated area where cells can constrict. Photo-activation was achieved with a 2.5 s pulse of 950 nm light by illuminating each area every 30 s. **(H)** Confocal image of the same embryo taken 22 min after the first pulse of light. CIBN::pmGFP is shown. (n=3 embryos). Scale bars, 10 µm.

Supplemental Movie Legends

Movie S1. Ventral Furrow Formation Dynamics in a Control Embryo, Related to Figure 2.
Time-lapse movie of a control embryo expressing only CIBN::pmGFP (white) during ventral furrow formation. The embryo was imaged with 488 nm light. Scale bar, 10 µm.

Movie S2. The Effects of Global Activation of CRY2-OCRL Recruitment to the Plasma Membrane on Ventral Furrow Formation, Related to Figure 2.
(A) Global activation of mCherry::CRY2-OCRL recruitment to the plasma membrane results in the inhibition of apical constriction and ventral furrow formation. Time-lapse movie of a photo-activated embryo co-expressing CIBN::pmGFP (not shown) and mCherry::CRY2-OCRL (white). CRY2-OCRL plasma membrane recruitment was activated with 488 nm laser illumination in the whole embryo, before ventral mesodermal cells started to constrict. Of note, the punctate fluorescence corresponds to clusters of CRY2-OCRL and CIBN-GFP at the plasma membrane, presumably caused by the tendency of CRY2 to oligomerize (Bugaj et al., 2013).
(B) Global activation of mCherry::CRY2-OCRL recruitment to the plasma membrane results in the reversion of ventral furrow formation. Time-lapse movie of a photo-activated embryo co-expressing CIBN::pmGFP (not shown) and mCherry::CRY2-OCRL (white). CRY2-OCRL plasma membrane recruitment was activated with 488 nm laser illumination in the whole embryo, after ventral mesodermal cells had started to constrict. Scale bars, 10 µm.

Movie S3. Ventral Furrow Formation Progresses Normally in Non-Activated *sktl*$^{+/-}$ and *zip*$^{+/-}$ Heterozygous Embryos, Related to Figure 3.
(A) Ventral furrow formation in an embryo co-expressing CIBN::pmGFP and mCherry::CRY2-OCRL in a *sktl*$^{+/-}$ background progresses normally. Time-lapse movie of an embryo co-expressing CIBN::pmGFP (not shown) and mCherry::CRY2-OCRL (white). The embryo is heterozygous for a *sktl* loss of function allele. The embryo was imaged with 561 nm light only.
(B) Ventral furrow formation in an embryo co-expressing CIBN::pmGFP and mCherry::CRY2-OCRL in a *zip*$^{+/-}$ background progresses normally. Time-lapse movie of an embryo co-expressing CIBN::pmGFP (not shown) and mCherry::CRY2-OCRL (white). The embryo is heterozygous for a *zip* loss of function allele. The embryo was imaged with 561 nm light only. Scale bars, 10 µm.

Movie S4. Local Activation of mCherry::CRY2-OCRL Recruitment to the Plasma Membrane Results in the Inhibition of Ventral Furrow Formation, Related to Figure 4.
Time-lapse movie of an embryo co-expressing CIBN::pmGFP (not shown) and mCherry::CRY2-OCRL (white). CRY2-OCRL plasma membrane recruitment was activated with two-photon laser illumination (λ = 950 nm) only in ventral mesodermal cells (red box), before the beginning of ventral furrow formation. Of note, the movement of the mesoderm to the right side (posterior) is presumably caused by pulling forces exerted by posterior midgut invagination and/or by the posterior flow of cells during germ-band extension. Scale bar, 10 µm.

Movie S5. Modulation of Apical Constriction at Different Laser Powers, Related to Figure 5.
(A) Effect of local activation of mCherry::CRY2-OCRL recruitment to the plasma membrane with 3.0 mW laser power. Confocal movie of an embryo co-expressing CIBN::pmGFP (not shown) and mCherry::CRY2-OCRL (white). CRY2-OCRL plasma membrane recruitment was activated only in a sub-group of ventral mesodermal cells (red box) with two-photon laser illumination (λ = 950 nm, laser power = 3.0 mW).
(B) Effect of local activation of mCherry::CRY2-OCRL recruitment to the plasma membrane with 1.5 mW laser power. Confocal movie of an embryo co-expressing CIBN::pmGFP (not shown) and mCherry::CRY2-OCRL (white). CRY2-OCRL plasma membrane recruitment was activated only in a sub-group of ventral mesodermal cells (red box) with two-photon laser illumination (λ = 950 nm, laser power = 1.5 mW).
(C) Effect of local activation of mCherry::CRY2-OCRL recruitment to the plasma membrane with 0.7 mW laser power. Confocal movie of an embryo co-expressing CIBN::pmGFP (not shown) and mCherry::CRY2-OCRL (white). CRY2-OCRL plasma membrane recruitment was activated only in a sub-group of ventral mesodermal cells (red box) with two-photon laser illumination (λ = 950 nm, laser power = 0.7 mW). Scale bars, 10 µm.

Movie S6. Local Inhibition of Apical Constriction at the Anterior or Posterior Ends of the Ventral Furrow Tissue, Related to Figure 6.
(A) Effect of activation of mCherry::CRY2-OCRL recruitment to the plasma membrane at the anterior end of the ventral furrow tissue. Confocal movie of an embryo co-expressing CIBN::pmGFP (not shown) and mCherry::CRY2-OCRL (white). CRY2-OCRL plasma membrane recruitment was activated only in a sub-group of ventral mesodermal cells (red box) with 950 nm light.
(B) Effect of activation of mCherry::CRY2-OCRL recruitment to the plasma membrane at the posterior end of the ventral furrow tissue. Confocal movie of an embryo co-expressing CIBN::pmGFP (not shown) and mCherry::CRY2-OCRL (white). CRY2-OCRL plasma membrane recruitment was activated only in a sub-group of ventral mesodermal cells (red box) with 950 nm light. Scale bars, 10 μm.

Movie S7. Local Inhibition of Apical Constriction in the Middle of the Ventral Furrow Tissue, Related to Figure 6.
(A) Effect of activation of mCherry::CRY2-OCRL recruitment to the plasma membrane in a sub-group of 50 cells in the middle of the ventral furrow tissue. Confocal movie of an embryo co-expressing CIBN::pmGFP (not shown) and mCherry::CRY2-OCRL (white). CRY2-OCRL plasma membrane recruitment was activated only in a sub-group of ventral mesodermal cells (red box) with 950 nm light.
(B) Effect of activation of mCherry::CRY2-OCRL recruitment to the plasma membrane in a sub-group of 25 cells in the middle of the furrow tissue. Confocal movie of an embryo co-expressing CIBN::pmGFP (not shown) and mCherry::CRY2-OCRL (white). CRY2-OCRL plasma membrane recruitment was activated only in a sub-group of ventral mesodermal cells (red box) with 950 nm light. Scale bars, 10 μm.

Movie S8. The Impact of Tissue Geometry on Contractile Behavior, Related to Figure 7.
(A) The degree of a-p anisotropy of cell shape changes depends on the geometry of the ventral furrow tissue. Confocal movie of an embryo co-expressing CIBN::pmGFP (not shown) and mCherry::CRY2-OCRL (white). CRY2-OCRL plasma membrane recruitment was activated with two-photon laser illumination (λ = 950 nm) in two groups of ventral mesodermal cells, one at the posterior and one at the anterior region of the furrow primordium (red boxes).
(B) The degree of a-p anisotropy of cell shape changes depends on the geometry of the ventral furrow tissue. Confocal movie of an embryo co-expressing CIBN::pmGFP (not shown) and mCherry::CRY2-OCRL (white). CRY2-OCRL plasma membrane recruitment was activated with two-photon laser illumination (λ = 950 nm) in two groups of ventral mesodermal cells, one at the posterior and one at the anterior region of the furrow primordium (red boxes). Scale bars, 10 μm.

Movie S9. Ventral furrow formation depends on the number of cells that can constrict, Related to Figure 7.
Confocal movie of an embryo co-expressing CIBN::pmGFP (not shown) and mCherry::CRY2-OCRL (white). CRY2-OCRL plasma membrane recruitment was activated with two-photon laser illumination (λ = 950 nm) in two groups of ventral mesodermal cells, one at the posterior and one at the anterior region of the furrow primordium (red boxes). Scale bar, 10 μm.

Supplemental Experimental Procedures

Cloning

Plasmids encoding CIBN::pmGFP and CRY2$_{\mathrm{PHR}}$ were purchased from Addgene (plasmid no. 26867 and 26866, respectively). CIBN::pmGFP and CRY2$_{\mathrm{PHR}}$ were PCR-amplified using gene-specific primers. To generate a non-tagged version of CIBN localizing to the plasma membrane, the sequence encoding CIBN was PCR-amplified from Addgene plasmid no. 26867 and fused C-terminally to the CaaX sequence (KKKKKKSKTKCVIM) of human KRas4B through overlap extension PCR. The catalytic domain (residues 99-519) of OCRL was PCR-amplified from *Drosophila melanogaster* cDNA using gene-specific primers and fused downstream of CRY2$_{\mathrm{PHR}}$. To generate a fluorescently tagged version of CRY2$_{\mathrm{PHR}}$-OCRL, mCherry fused C-terminally to a GAGA linker was cloned upstream of CRY2-OCRL. All constructs were cloned into the pPW vector (Drosophila Genomics Resource Center, Bloomington, IN) using the Gateway cloning system (Life Technologies) according to standard procedures.

Fly genetics

Transgenic flies were obtained by standard methods and all stocks were maintained at 22°C. Standard genetic crosses were used to generate flies having the following genotypes:

To test for the effect of CRY2-OCRL plasma membrane recruitment on morphogenesis,

w[*]; P[w+, UASp>CIBN::pmGFP]/P[w+, matαTub>Gal4::VP16]; P[w+, UASp>mCherry::CRY2-OCRL]/ P[w+, matαTub>Gal4::VP16].

To test for PI(4,5)P$_2$ depletion,

P[w+, UASp>pmCIBN]; P[w+, btl>Gal4, UAS>PLC-PH::GFP]/ P[w+, matαTub>Gal4::VP16]; P[w+, UASp>mCherry::CRY2-OCRL]/ P[w+, matαTub>Gal4::VP16].

To test for Moesin depletion,

P[w+, UASp>CRY2-OCRL]; P[w+, UASp>CIBN::pmGFP]/ P[w+, matαTub>Gal4::VP16]; P{w[+mC]=sChMCA}31/P[w+, matαTub>Gal4::VP16].

To test for the dark state activity of CRY2-OCRL and CIBN::pmGFP in a heterozygous *zip* mutant background,

w[*]; cn[1] bw[1] sp[1] zip[1]/P[w+, UASp>CIBN::pmGFP]; P[w+, UASp>mCherry::CRY2-OCRL]/ P[w+, matαTub>Gal4::VP16].

To test for the dark state activity of CRY2-OCRL and CIBN::pmGFP in a heterozygous *sktl* mutant background,

w[*]; sktl[Delta20]/P[w+, UASp>CIBN::pmGFP]; P[w+, UASp>mCherry::CRY2-OCRL]/ P[w+, matαTub>Gal4::VP16].

All crosses were kept in the dark at 22°C. Flies of the desired genotype were maintained in cages with apple juice plates covered with yeast paste and kept in the dark at 18°C for embryo collection.

Fly stocks

w[*];P[w+, btl>Gal4, UAS>PLC-PH::GFP]; . (kindly provided by M. Leptin, EMBL Heidelberg, Germany) The PH domain (residues 11–170) of human PLCδ1 was fused C-terminally to GFP and cloned into a CaSpeR-based transformation vector.

w[*]; P[w+, matαTub>Gal4::VP16]; [w+, mat αTub>Gal4::VP16]. (kindly provided by E. Wieschaus, Princeton University, Princeton, NJ) The maternal tubulin promoter was cloned upstream of the sequence encoding Gal4::VP16 into a CaSpeR-based vector.

w[1118]; ; P{w[+mC]=sChMCA}31. (Bloomington Drosophila Stock Center, Bloomington, IN) The actin binding domain of Moesin, driven constitutively with the sqh promoter, was fused to mCherry and cloned into a CaSpeR-based vector (Abreu-Blanco et al., 2011).

y[1] w[*]; sktl[Delta20]/CyO, P{ry[+t7.2]=ftz/lacB}E3. (Bloomington Drosophila Stock Center, Bloomington, IN) Loss of function *sktl* allele (Hassan et al., 1998).

cn[1] bw[1] sp[1] zip[1]/CyO (Bloomington Drosophila Stock Center, Bloomington, IN) Amorphic *zip* allele (Tearle and Nusslein-Volhard, 1987).

w[*]; P[w+, UASp>CIBN::pmGFP]/CyO; Sb/TM3, Ser. (this paper) The N-terminal end (residues 1-170) of CIB1 protein, fused to a version of enhanced GFP that localizes to the plasma membrane through a CaaX anchor, was cloned into pPW vector (Drosophila Genomics Resource Center, Bloomington, IN).

w[*]; If/CyO; P[w+, UASp>mCherry::CRY2-OCRL]/TM3, Ser. (this paper) The PHR domain of CRY2, fused to *Drosophila melanogaster* inositol polyphosphate 5-phosphatase OCRL, was fused downstream of mCherry and spaced with a GAGA linker. The resulting chimeric protein was cloned into pPW vector (Drosophila Genomics Resource Center, Bloomington, IN).

P[w+, UASp>pmCIBN]/FM6; ;Sb/TM6B, Tb. (this paper) The N-terminal end (residues 1-170) of CIB1 protein, fused to the CaaX anchor of human Kras4B, was cloned into pPW vector (Drosophila Genomics Resource Center, Bloomington, IN).

P[w+, UASp>CRY2-OCRL]/FM7; Sco/CyO; . (this paper) The PHR domain of CRY2 was fused to *Drosophila melanogaster* inositol polyphosphate 5-phosphatase OCRL and cloned into pPW vector (Drosophila Genomics Resource Center, Bloomington, IN).

Live imaging and Photo-activation Protocol

To avoid unwanted photo-activation, embryos were staged and mounted using a standard upright microscope equipped with a 10x objective (Carl Zeiss) in a dark room. The microscope light source was replaced with a lamp (Tween Light) equipped with a yellow 11W incandescence bulb (Décor Color Yellow 11, Osram). Embryos were visualized using a thin layer of Halocarbon oil 27 (Sigma-Aldrich) and those at the right developmental stage were selected and dechorionated with 100% sodium hypochlorite solution for 2 minutes, washed with water, and mounted on a 35 mm glass bottom dish having a 14 mm microwell (coverslip thickness: 0.16-0.19 mm) (MatTek corporation).

Embryos were covered with PBS and imaged at 20°C with a Zeiss LSM 780 NLO confocal microscope (Carl Zeiss) equipped with a 561 diode laser, an argon laser and a two-photon laser (Chameleon; Coherent, Inc.). Since bright field illumination could result in unwanted photo-activation, the microscope light source was covered with a Deep Amber lighting filter (Cabledelight, Ltd). mCherry was excited with λ = 561 nm at a laser intensity of 50.8 μW, measured 1 cm from the objective. EGFP was excited with λ = 488 nm at a laser intensity of 6.9 μW, measured 1 cm from the objective. Photo-activation was tested with λ = 458 nm, 488 nm, 514 nm (one-photon illumination) and with λ = 900 nm, 950 nm and 1000 nm (two-photon illumination) by illuminating a single z-stack 5 μm from the apical surface for a total scan time of 1 s. Between two consecutive photo-activations there was an interval of 30 s and the acquisition of the mCherry channel at 561 nm. The laser intensities used were: 6.1 μW (458 nm), 6.9 μW (488 nm), 10.6 μW (514 nm), 6.5 mW (900 nm), 3.0 mW (950 nm), 1.03 mW (1000 nm). The laser intensities were measured 1 cm from the objective and corresponded to the ones normally used for optical imaging of fluorescent proteins.

Once established the most effective wavelength to trigger CRY2-OCRL recruitment to the plasma membrane, photo-activation with one-photon illumination was achieved with λ = 488 nm by illuminating a single z-stack 5 μm from the apical surface for a total scan time of 1 s. Between two consecutive excitations there was an interval of 30 s and the acquisition of the mCherry channel at 561 nm. The laser power used was equivalent to the one used for EGFP imaging (6.9 μW, measured 1 cm from the objective).

Photo-activation with two-photon illumination was achieved with λ = 950 nm by illuminating five z-stacks (each plane separated by 1 μm) for a total scan time of 2.5 s (~500 ms per plane). Laser powers used were 3.0 mW, 1.5 mW and 0.7 mW, measured 1 cm from the objective. Unless otherwise stated, local photo-activation experiments were achieved with a laser intensity of 3.0 mW. The first frame was acquired at 561 nm to image CRY2-OCRL prior to activation. Between two consecutive two-photon excitations there was an interval of 30 s and the acquisition of the mCherry channel at 561 nm.

To test for photo-activation at different depths, we illuminated for a total scan time of 2.5 s five consecutive z-stacks (each plane separated by 0.5 μm), with the central z-stack being taken at 5

μm, 10 μm, 20 μm and 80 μm from the apical surface. Two-photon activation was achieved as described before with a laser intensity of 3.0 mW (measured 1 cm from the objective).

Videos and images were acquired using a C-Apochromat 63x/NA 1.2 water immersion objective (Carl Zeiss), with the exception of images shown in Figure S1F-L, which were acquired with a 40x/NA 1.1 water immersion objective (Carl Zeiss). Confocal images are single z-stacks taken 5 μm below the apical cortex. Movies and corresponding still frames are sum projections of five z-stacks taken 5 μm below the apical cortex. In Figure S1F-L, confocal images are single z-stacks taken at the embryo cross-section (~80 μm from the apical cortex). Images were acquired using Zen 2011 software (Carl Zeiss). Images were processed with FIJI (Schindelin et al., 2012) and custom-written R software (see "Image Analysis and Quantification of Cell Features" section).

Image Analysis and Quantification of Cell Features

Images were opened and metadata extracted using the RBioFormats software package (https://github.com/aoles/RBioFormats). Images were processed in R using custom-written software that utilized the image processing toolbox EBImage (Pau et al., 2010). Each z-stack was first smoothed with a Gaussian filter of diameter ~1 μm. Membrane was identified using an adaptive thresholding algorithm that used a moving box of diameter ~2.5 μm. Pixel noise on the membrane mask was reduced by performing a closing morphological operation with a filter diameter of ~1 μm. To reduce the number of false positive (non-membrane) pixels, objects with an area smaller than 4 μm^2 were removed at this stage. The membrane mask was inverted to obtain masks for cytoplasmic areas, which were used as seeds in a voronoi-based segmentation algorithm to identify cell borders more precisely. Finally, we omitted objects that were below an area of 4 μm^2 or above 400 μm^2 to reduce the number of false positive cells.

Cell area, major axis, eccentricity, and angle with respect to the a-p axis were calculated for each z-stack separately using the computeFeatures function from the EBImage toolbox. To calculate cell anisotropy we first weighted the unit vector along the major axis of the cell by the cell eccentricity. We defined the first and second components of the resulting vector as the a-p and d-v anisotropy, respectively. This resulted in quantities between zero and one that were invariant with respect to isotropic rescaling of cell size. Since we were primarily interested in quantifying elongation along the a-p axis, we focused on the a-p anisotropy in the analysis.

The level of optogenetic activation of a cell was calculated by manually defining the membrane mask with the EGFP channel. Nuclei, which tended to be darker than true cytoplasmic regions, were also manually removed. We then quantified the level of optogenetic activation of each cell by calculating the log_2 ratio between the average membrane and average cytosol intensities.

A similar procedure was used to quantify the plasma membrane levels of $PH_{PLC\delta}$::GFP and Moesin::mCherry. Here, for each embryo we calculated the plasma membrane fluorescence as the ratio of the average membrane to average cytosol intensities at the beginning of photo-activation (T_0), and after 1 min (for $PH_{PLC\delta}$::GFP) or 4 min (for Moesin::mCherry) of photo-activation. We then calculated the log_2 ratio of the plasma membrane fluorescence intensity after photo-activation to the plasma membrane fluorescence intensity at T_0. Plots were generated using R package ggplot2 (http://ggplot2.org).

Supplemental References

Abreu-Blanco, M.T., Verboon, J.M., and Parkhurst, S.M. (2011). Cell wound repair in Drosophila occurs through three distinct phases of membrane and cytoskeletal remodeling. J Cell Biol *193*, 455-464.

Bugaj, L.J., Choksi, A.T., Mesuda, C.K., Kane, R.S., and Schaffer, D.V. (2013). Optogenetic protein clustering and signaling activation in mammalian cells. Nature methods *10*, 249-252.

Hassan, B.A., Prokopenko, S.N., Breuer, S., Zhang, B., Paululat, A., and Bellen, H.J. (1998). skittles, a Drosophila phosphatidylinositol 4-phosphate 5-kinase, is required for cell viability, germline development and bristle morphology, but not for neurotransmitter release. Genetics *150*, 1527-1537.

Pau, G., Fuchs, F., Sklyar, O., Boutros, M., and Huber, W. (2010). EBImage--an R package for image processing with applications to cellular phenotypes. Bioinformatics (Oxford, England) *26*, 979-981.

Schindelin, J., Arganda-Carreras, I., Frise, E., Kaynig, V., Longair, M., Pietzsch, T., Preibisch, S., Rueden, C., Saalfeld, S., Schmid, B., *et al.* (2012). Fiji: an open-source platform for biological-image analysis. Nat Methods *9*, 676-682.
