# Supplementary figures and images for: An Optogenetic Method to Modulate Cell Contractility during Tissue Morphogenesis
Source: Dev Cell. 2015 Dec 7;35(5):646–60. doi: 10.1016/j.devcel.2015.10.020 (PMC4683098; doi:10.1016/j.devcel.2015.10.020)

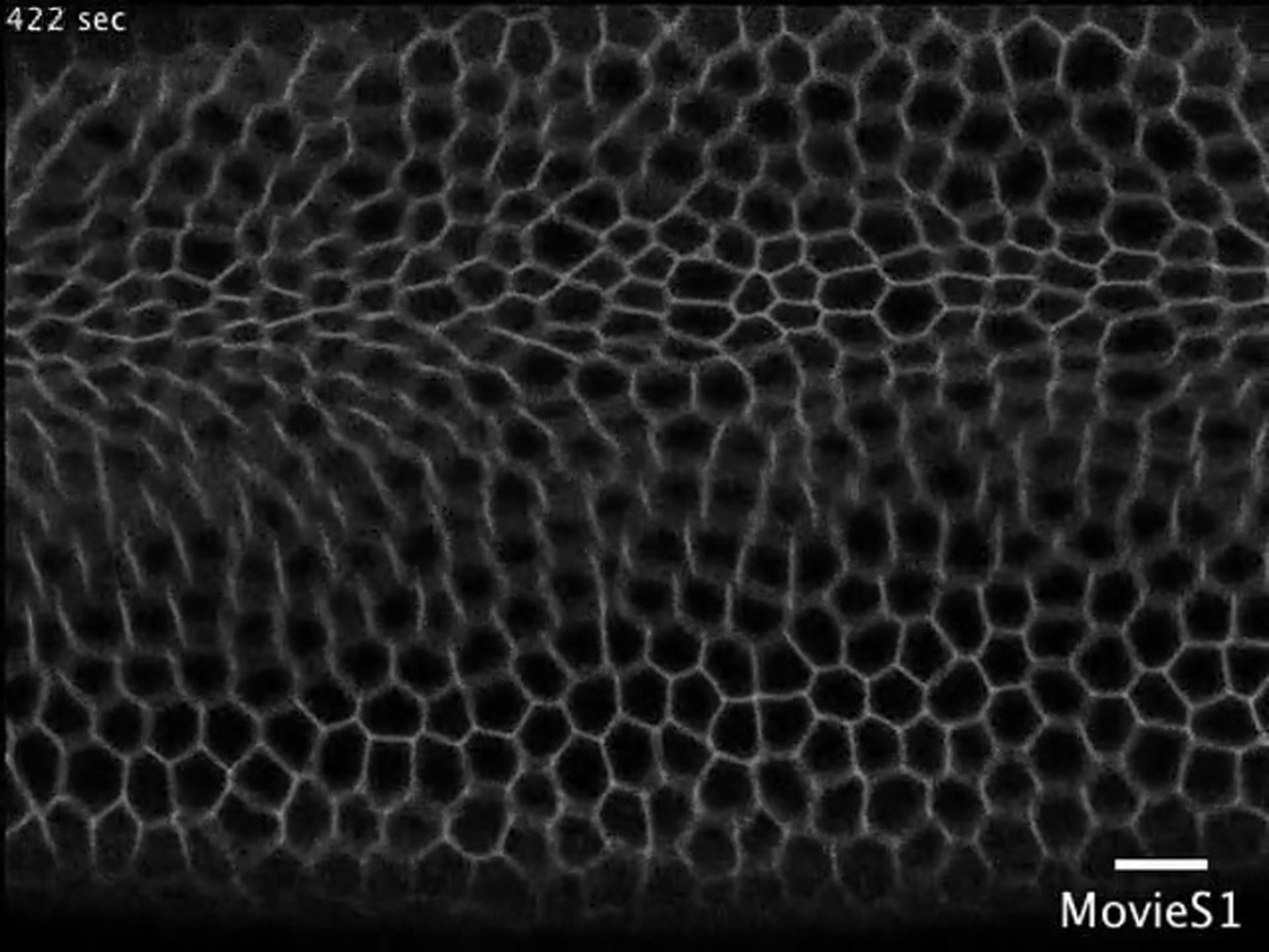

Supplement: Movie S1. Ventral Furrow Formation Dynamics in a Control Embryo, Related to Figure 2 [file mmc2.jpg]

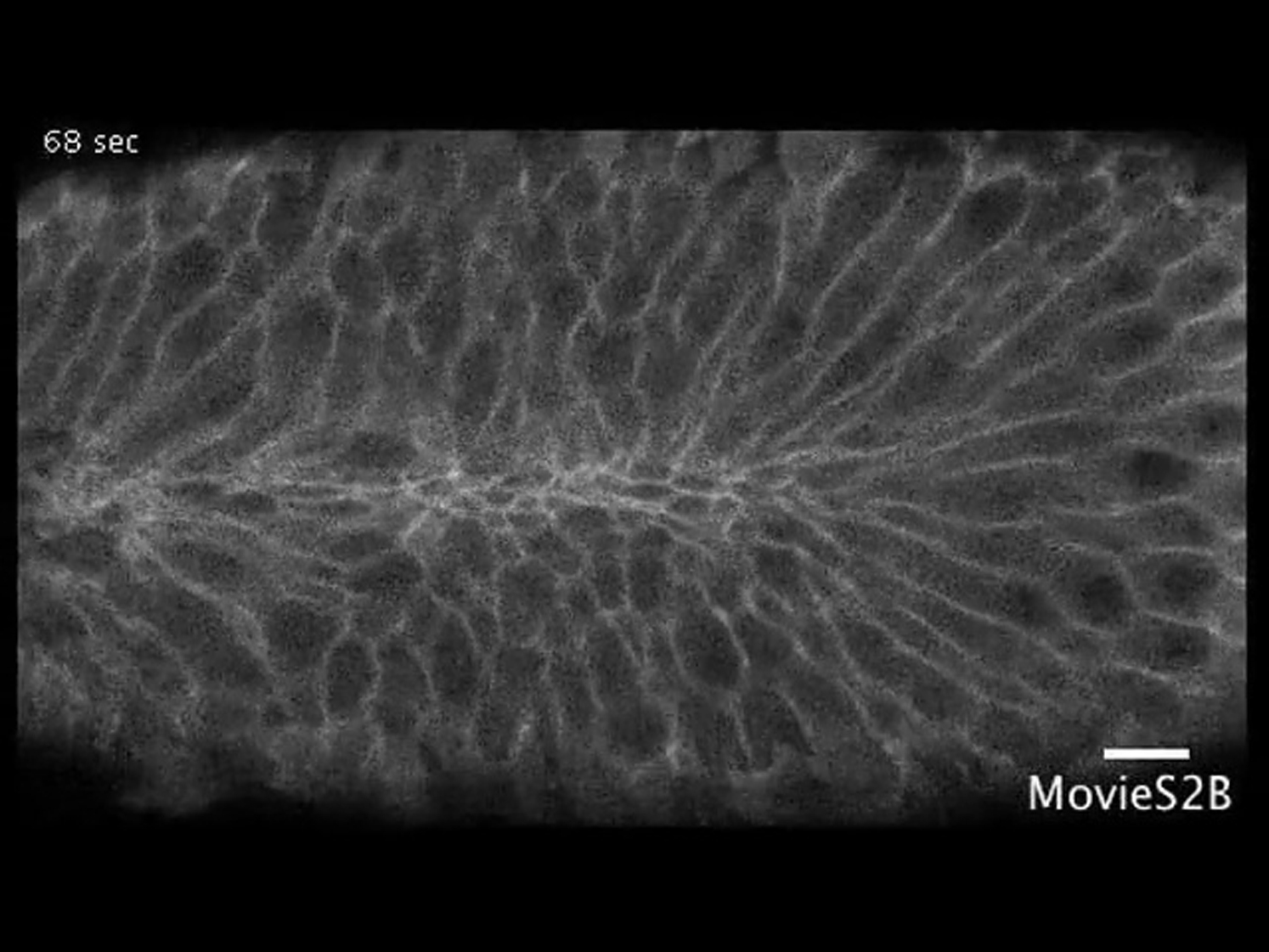

Supplement: Movie S2. The Effects of Global Activation of CRY2-OCRL Recruitment to the Plasma Membrane on Ventral Furrow Formation, Related to Figure 2 [file mmc3.jpg]

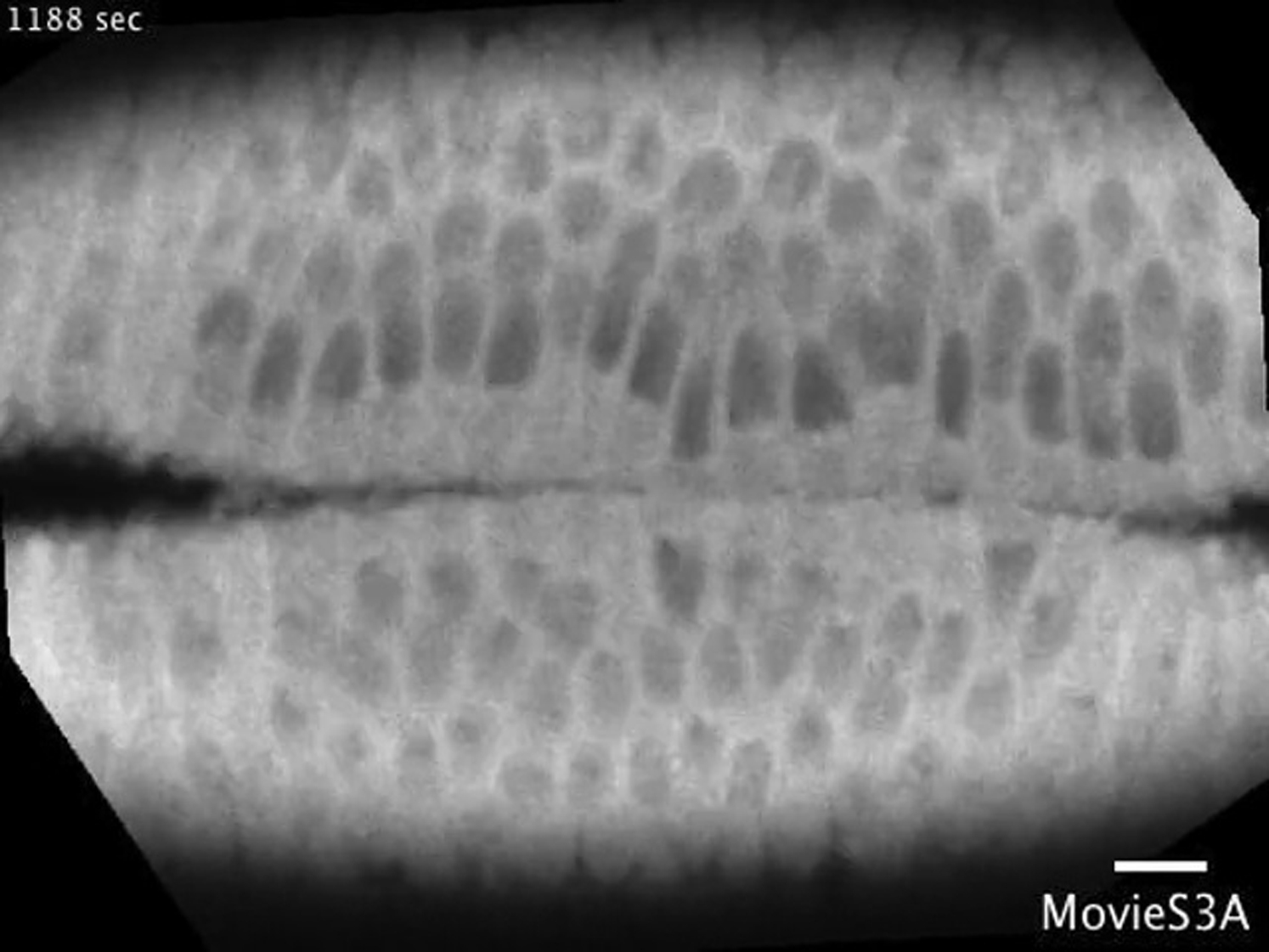

Supplement: Movie S3. Ventral Furrow Formation Progresses Normally in Non-activated sktl+/− and zip+/− Heterozygous Embryos, Related to Figure 3 [file mmc4.jpg]

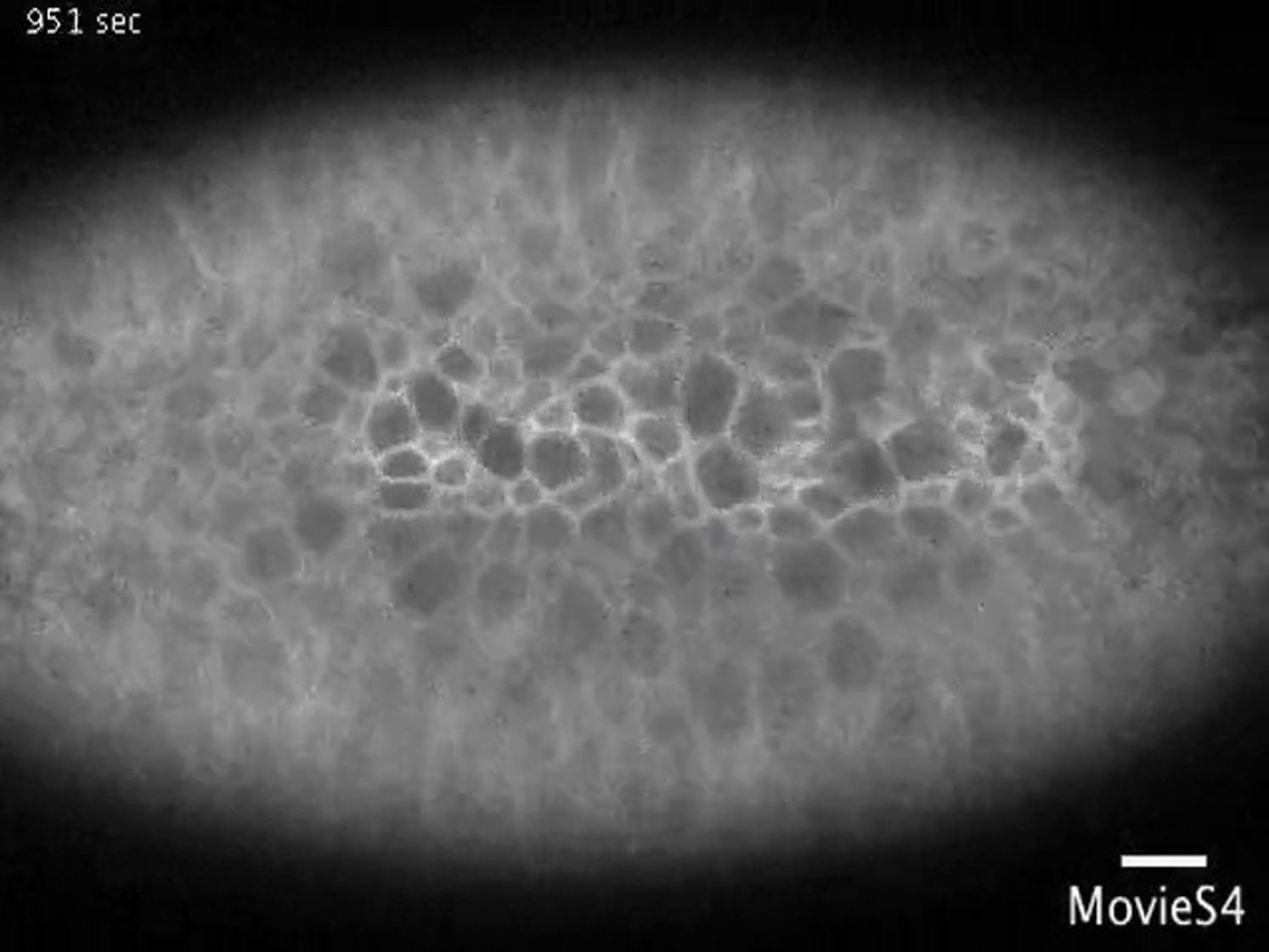

Supplement: Movie S4. Local Activation of mCherry::CRY2-OCRL Recruitment to the Plasma Membrane Results in the Inhibition of Ventral Furrow Formation, Related to Figure 4 [file mmc5.jpg]

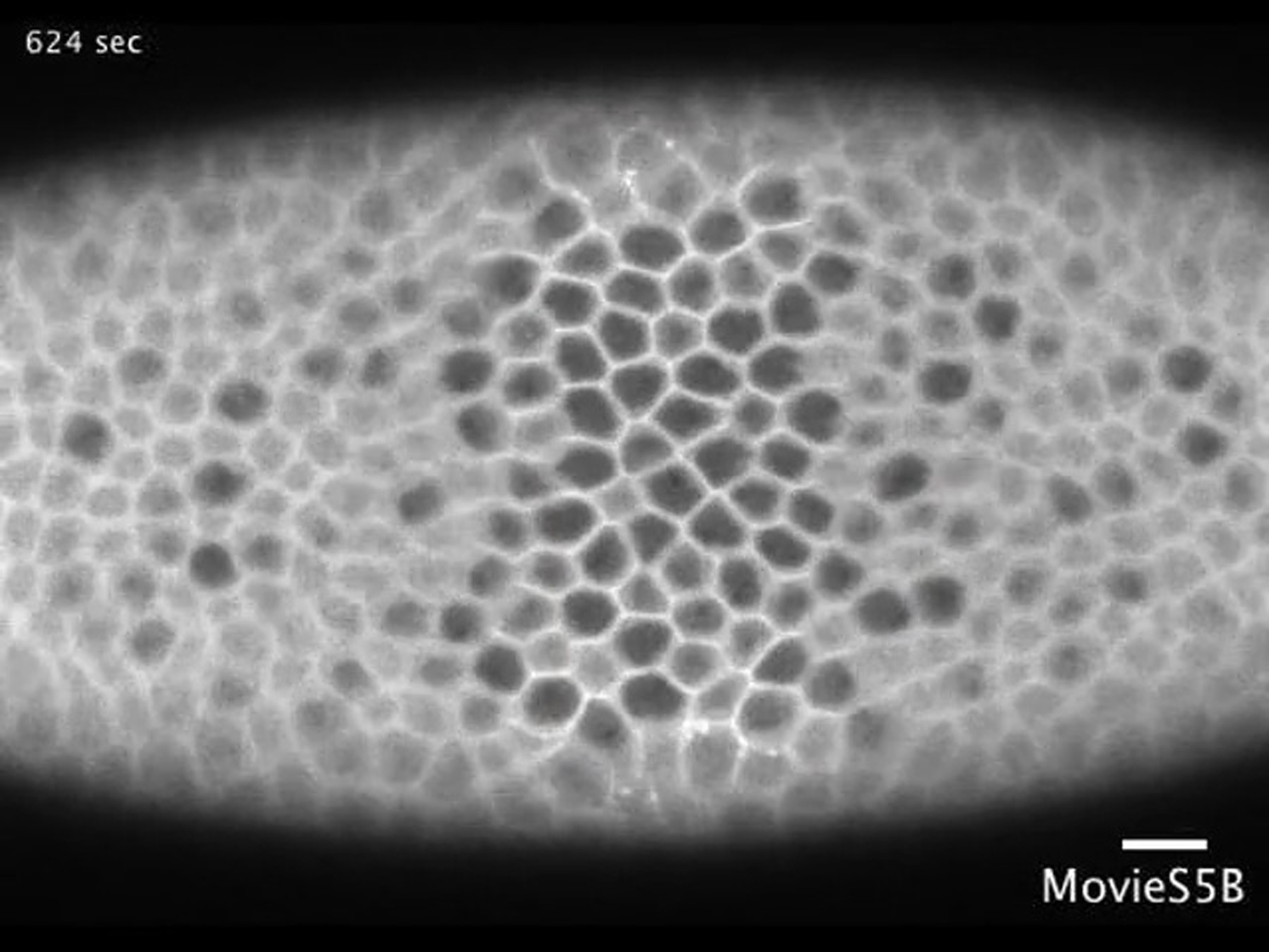

Supplement: Movie S5. Modulation of Apical Constriction at Different Laser Powers, Related to Figure 5 [file mmc6.jpg]

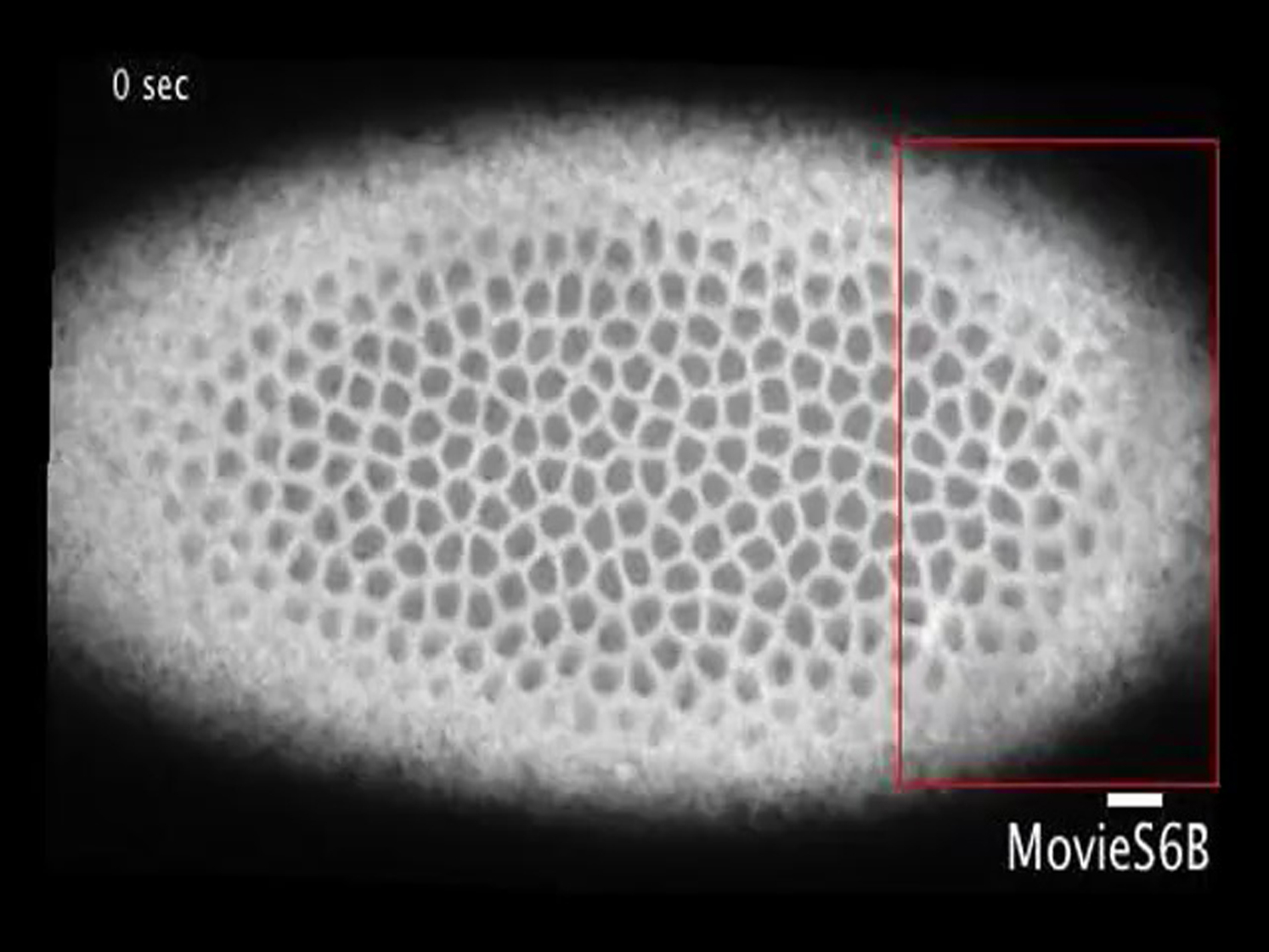

Supplement: Movie S6. Local Inhibition of Apical Constriction at the Anterior or Posterior Ends of the Ventral Furrow Tissue, Related to Figure 6 [file mmc7.jpg]

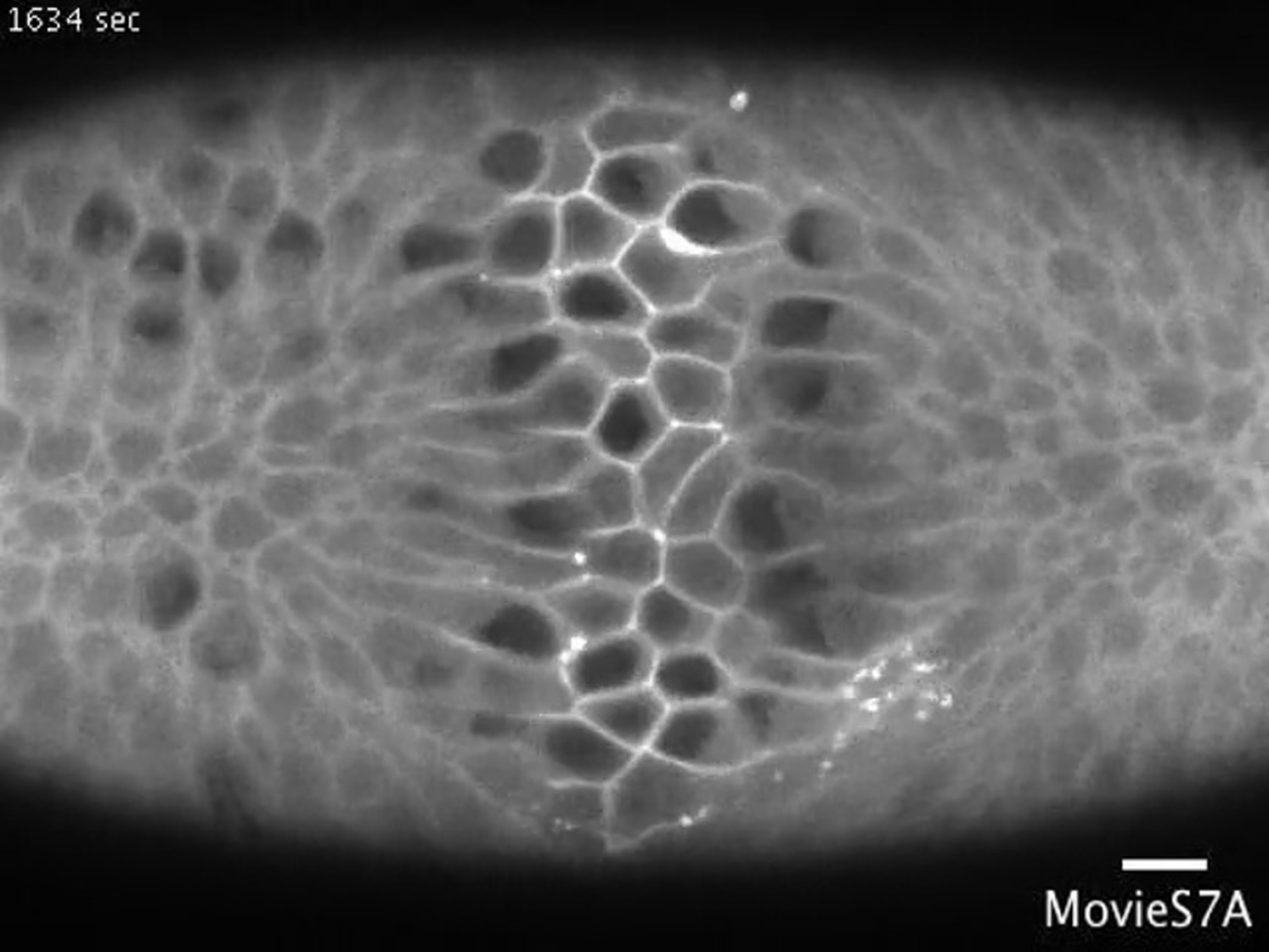

Supplement: Movie S7. Local Inhibition of Apical Constriction in the Middle of the Ventral Furrow Tissue, Related to Figure 6 [file mmc8.jpg]

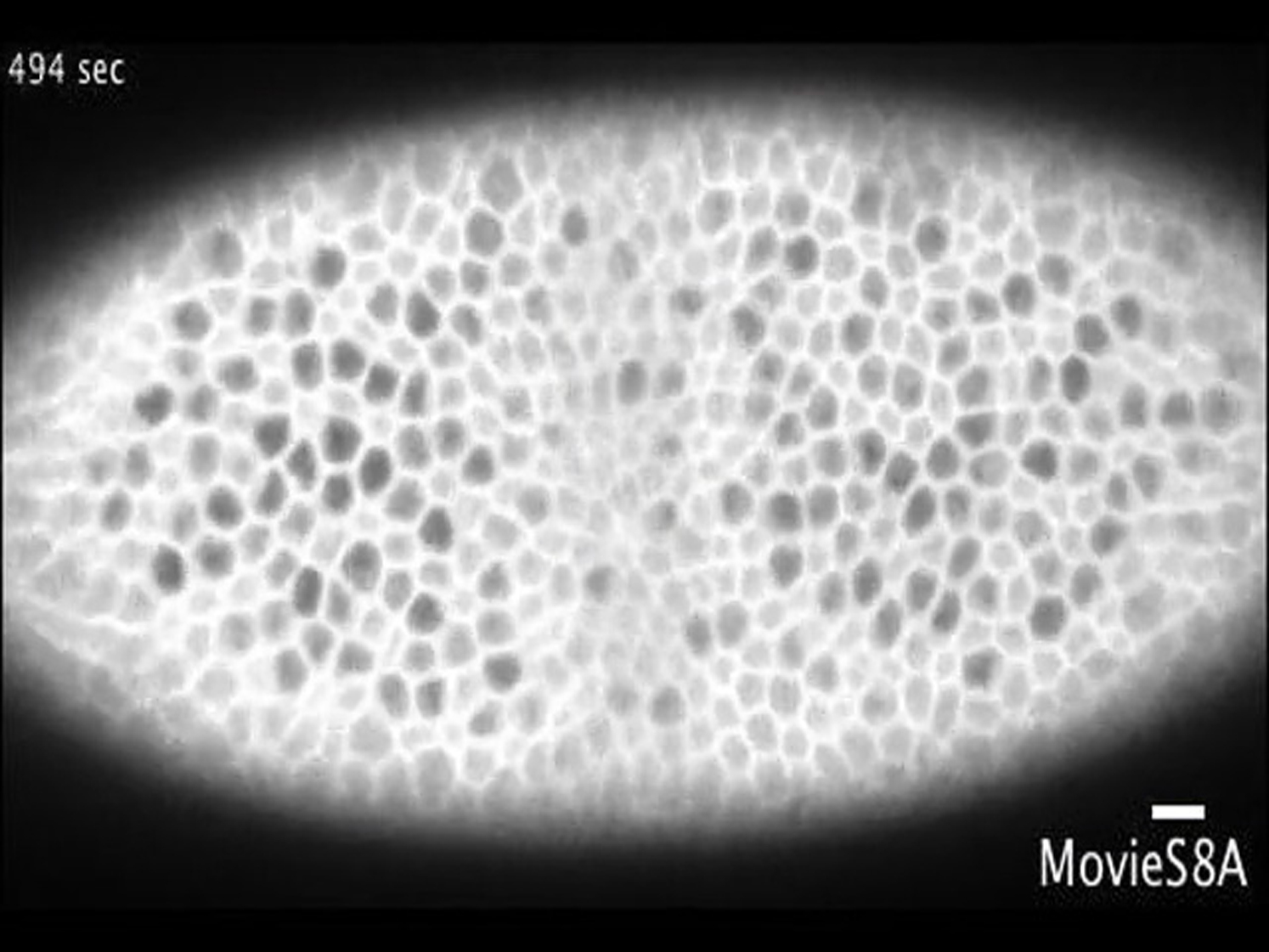

Supplement: Movie S8. The Impact of Tissue Geometry on Contractile Behavior, Related to Figure 7 [file mmc9.jpg]

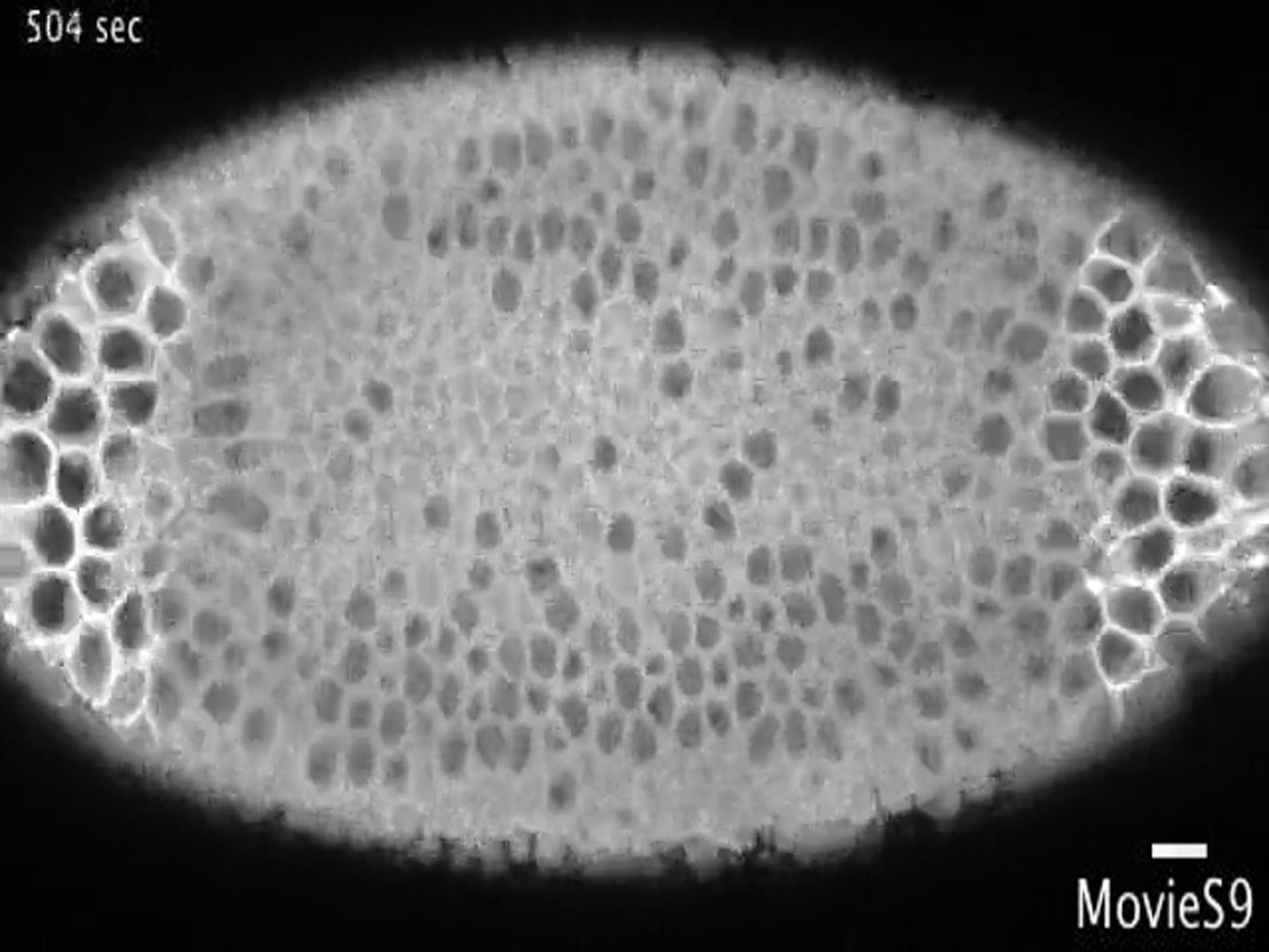

Supplement: Movie S9. Ventral Furrow Formation Depends on the Number of Cells that Can Constrict, Related to Figure 7 [file mmc10.jpg]
